# Supplementary material for: Targeted metagenomics reveals association between severity and pathogen co-detection in infants with respiratory syncytial virus
Source: Nat Commun. 2024 Mar 16;15:2379. doi: 10.1038/s41467-024-46648-3 (PMC10944482; doi:10.1038/s41467-024-46648-3)
Supplement: Supplementary file 1 — Supplementary Information [file 41467_2024_46648_MOESM1_ESM.pdf]

Supplementary Information

Targeted metagenomics reveals association between severity and pathogen co-detection in infants with respiratory syncytial virus

Gu-Lung Lin[1,2,†], Simon B Drysdale[1,2,3], Matthew D Snape[1,2], Daniel O'Connor[1,2], Anthony Brown[4], George MacIntyre-Cockett[5], Esther Mellado-Gomez[5,6], Mariateresa de Cesare[5,7], M Azim Ansari[4,5], David Bonsall[5,8], James E Bray[9], Keith A Jolley[9], Rory Bowden[5,10,11], Jeroen Aerssens[12], Louis Bont[13,14], Peter J M Openshaw[15], Federico Martinon-Torres[16,17,18], Harish Nair[19,20], Tanya Golubchik[8,21,22,†], Andrew J Pollard[1,2,22], RESCEU Consortium*

[1]Oxford Vaccine Group, Department of Paediatrics, University of Oxford, Oxford, UK
[2]NIHR Oxford Biomedical Research Centre, Oxford, UK
[3]Centre for Neonatal and Paediatric Infection, Institute for Infection and Immunity, St George's, University of London, London, UK
[4]Peter Medawar Building for Pathogen Research, University of Oxford, Oxford, UK
[5]Wellcome Centre for Human Genetics, University of Oxford, Oxford, UK
[6]Wellcome Sanger Institute, Hinxton, UK
[7]Human Technopole, Milan, Italy
[8]Big Data Institute, Nuffield Department of Medicine, University of Oxford, Oxford, UK
[9]Department of Biology, University of Oxford, Oxford, UK
[10]The Walter and Eliza Hall Institute of Medical Research, Parkville, VIC, Australia
[11]Department of Medical Biology, University of Melbourne, Melbourne, VIC, Australia
[12]Translational Biomarkers, Infectious Diseases Therapeutic Area, Janssen Pharmaceutica NV, Beerse, Belgium
[13]Department of Pediatrics, Wilhelmina Children's Hospital, University Medical Center Utrecht, Utrecht, Netherlands
[14]ReSViNET Foundation, Zeist, Netherlands
[15]National Heart and Lung Institute, Imperial College London, London, UK
[16]Translational Pediatrics and Infectious Diseases, Pediatrics Department, Hospital Clínico Universitario de Santiago de Compostela, Santiago de Compostela, Spain
[17]Genetics, Vaccines, Infectious Diseases and Pediatrics Research Group (GENVIP), Instituto de Investigación Sanitaria de Santiago, University of Santiago de Compostela, Santiago de Compostela, Spain
[18]Centro de Investigación Biomédica en Red de Enfermedades Respiratorias (CIBERES), Instituto de Salud Carlos III, Madrid, Spain
[19]Centre for Global Health, Usher Institute, Edinburgh Medical School, University of Edinburgh, Edinburgh, UK
[20]MRC/Wits Rural Public Health and Health Transitions Research Unit (Agincourt), School of Public Health, Faculty of Health Sciences, University of the Witwatersrand, Johannesburg, South Africa
[21]Sydney Infectious Diseases Institute, School of Medical Sciences, Faculty of Medicine and Health, University of Sydney, Sydney, Australia
[22]These authors jointly supervised this work: Tanya Golubchik, Andrew J Pollard.
*See next page for a full list of members and their affiliations.
[†]Corresponding authors: Gu-Lung Lin, Tanya Golubchik
E-mails: gulung.lin.oxford@gmail.com; tanya.golubchik@sydney.edu.au

RESCEU Consortium

Harish Nair, Harry Campbell, Steve Cunningham (University of Edinburgh); Philippe Beutels (Universiteit Antwerpen); Louis Bont, Joanne Wildenbeest, Debby Bogaert (University Medical Centre Utrecht); Andrew J Pollard, Matthew D Snape, Simon B Drysdale, Gu-Lung Lin, Daniel O'Connor, Elizabeth Clutterbuck, Joseph McGinley, Hannah Robinson (University of Oxford); Peter J M Openshaw, Ryan Thwaites, Dexter Wiseman (Imperial College London); Federico Martinon-Torres, Alberto Gómez-Carballa, Carmen Rodriguez-Tenreiro, Irene Rivero-Calle, Ana Dacosta-Urbieta, Sara Pischedda (Servicio Galego de Saude); Terho Heikkinen (University of Turku and Turku University Hospital); Adam Meijer (National Institute for Public Health and the Environment); Thea K Fischer (Statens Serum Institut); Maarten van den Berge (University of Groningen); Carlo Giaquinto (PENTA Foundation); Michael Abram (AstraZeneca); Kena Swanson (Pfizer); Bishoy Rizkalla (GSK); Charlotte Vernhes, Scott Gallichan (Sanofi Pasteur); Jeroen Aerssens, Deniz Öner (Janssen); Veena Kumar (Novavax); Eva Molero (Team-It Research).

Supplementary Table 1 | Number of RSV-infected infants from each country and each season (N = 440).

	Longitudinal birth cohort study (N = 146)	Infant cross-sectional study (N = 294)	Total (N = 440)
2017–18	13	57	70
Spain	10	0	10
United Kingdom	0	6	6
Netherlands	3	51	54
2018–19	41	122	163
Spain	10	10	20
United Kingdom	12	75	87
Netherlands	19	37	56
2019–20	92	115	207
Spain	32	15	47
United Kingdom	18	58	76
Netherlands	42	42	84
All periods (2017–20)	146	294	440
Spain	52	25	77
United Kingdom	30	139	169
Netherlands	64	130	194

Supplementary Table 2 | Characteristics of RSV-infected infants by age (N = 431).[a]

	<3 mo (N = 169)	3 to <6 mo (N = 117)	6 to <12 mo (N = 145)	P value
**Demographic features**				
Gestational age				
Median (IQR) — wk	39.3 (38.0–40.1)	40.0 (39.0–40.7)	39.7 (38.7–40.7)	7.3×10^{-4}
Distribution				0.399
<32 wk	4/168 (2)	1/115 (1)	3/144 (2)	
32 to <37 wk	10/168 (6)	2/115 (2)	5/144 (3)	
≥37 wk	154/168 (92)	112/115 (97)	136/144 (94)	
Female sex	66 (39)	51 (44)	75 (52)	0.077
Comorbidity	18 (11)	5 (4)	14 (10)	0.142
**Virological features**				
RSV-A[b]	79/165 (48)	66/114 (58)	74/143 (52)	0.442
Peak RSV read count — total no.	166	114	141	
Mean ± SD — $\log_{10}$	3.8 ± 1.1	4.2 ± 1.0	4.1 ± 1.0	0.081[c]
**Clinical features[d]**				
ReSVinet score				
Mean ± SD	9.6 ± 4.9	6.7 ± 4.5	5.9 ± 3.3	1.7×10^{-6}
Distribution				5.5×10^{-5}
0–7	63/166 (38)	74/113 (65)	102/140 (73)	
8–13	60/166 (36)	28/113 (25)	34/140 (24)	
14–20	43/166 (26)	11/113 (10)	4/140 (3)	
Fever	33/166 (20)	39/113 (35)	70/140 (50)	3.6×10^{-8}
Hospitalisation	130/167 (78)	47/104 (45)	42/132 (32)	2.5×10^{-9}
PICU admission	57/167 (34)	15/104 (14)	5/132 (4)	4.1×10^{-6}
Respiratory support	116/157 (74)	36/92 (39)	27/122 (22)	4.4×10^{-10}
Mechanical ventilation	53/157 (34)	10/92 (11)	3/122 (2)	1.4×10^{-6}

[a] Two participants without available age information were excluded from this table. Unless otherwise specified, data are shown as number/total number (%) or number (%) if there is no missing value. Percentages may not total 100 due to rounding. For demographic features, Kruskal–Wallis tests were used to compare continuous variables between the groups; two-sided chi-square tests with Yates' correction or two-sided Fisher's exact tests were used to compare categorical variables between the groups, whichever is appropriate. For virological and clinical features, likelihood-ratio tests were used to evaluate the effect of age on the goodness of fit of the models.

[b] Nine participants with both RSV subgroups A and B identified were excluded from this comparison. Two-sided multivariable logistic regression was used to adjust for sampling season.

[c] Two-sided multiple linear regression was used to adjust for the duration between symptom onset and sampling.

[d] Multiple linear regression, ordered logistic regression, or multivariable logistic regression (all two-sided) was used to adjust for covariates, depending on the type of the response (dependent) variable. Covariates included gestational age, sex, comorbidity, sampling season and country, study cohort, RSV subgroup, and peak RSV read count along with the duration between symptom onset and sampling. Models with different combinations of the covariates were tested, and the model with the lowest Akaike information criterion (AIC) was selected.

Abbreviations: *IQR* interquartile range, *PICU* paediatric intensive care unit, *RSV* respiratory syncytial virus, *SD* standard deviation.

Supplementary Table 3 | Incidences of the two RSV subgroups in each country and season (N = 433).

	RSV-A (N = 220)	RSV-B (N = 204)	Mixed[a] (N = 9)
2017–18	19	50	1
Spain	0	10	0
United Kingdom	4	2	0
Netherlands	15	38	1
2018–19	68	89	4
Spain	13	7	0
United Kingdom	40	45	2
Netherlands	15	37	2
2019–20	133	65	4
Spain	29	18	0
United Kingdom	45	29	0
Netherlands	59	18	4

[a] There was one case of plausible contamination: a nearly complete RSV-A genome and ~9% of an RSV-B genome were recovered from one of the nine samples. This partial RSV-B genome segment was nearly identical to part of the RSV-B genome recovered from another sample collected in the same season but a different country with a higher viral load (3.1×10^4 vs. 1.6×10^8 copies/mL). These two samples were located adjacent on the sequencing plate.

Supplementary Table 4 | Characteristics of the RSV-infected infants by RSV subgroup (N = 424).[a]

	RSV-A (N = 220)	RSV-B (N = 204)	P value	Q value
**Demographic features**				
Age				
Median (IQR) — mo	4.3 (1.9–7.3)	3.8 (1.7–7.5)	0.446	
Distribution — no./total no. (%)			0.258	
<3 mo	79/219 (36)	86/203 (42)		
3 to <6 mo	66/219 (30)	48/203 (24)		
6 to <12 mo	74/219 (34)	69/203 (34)		
Gestational age				
Median (IQR) — wk	39.9 (38.5–40.6)	39.6 (38.6–40.3)	0.501	
Distribution — no./total no. (%)			0.508	
<32 wk	3/215 (1)	5/203 (2)		
32 to <37 wk	10/215 (5)	6/203 (3)		
≥37 wk	202/215 (94)	192/203 (95)		
Female sex — no./total no. (%)	95/219 (43)	93/203 (46)	0.686	
Comorbidity — no./total no. (%)	16/220 (7)	20/203 (10)	0.438	
**Virological features**				
Peak RSV read count — total no.	214	199		
Mean ± SD — $\log_{10}$	4.1 ± 0.9	3.9 ± 1.1	0.067[b]	
**Clinical features[c]**				
ReSVinet score				
Mean ± SD	7.4 ± 4.2	7.6 ± 5.0	0.457	0.639
Distribution — no./total no. (%)			0.783	0.783
0–7	127/216 (59)	110/195 (56)		
8–13	64/216 (30)	54/195 (28)		
14–20	25/216 (12)	31/195 (16)		
Fever — no./total no. (%)	80/216 (37)	58/195 (30)	0.061	0.214
Hospitalisation — no./total no. (%)	115/205 (56)	97/190 (51)	0.037	0.214
PICU admission — no./total no. (%)	31/205 (15)	42/190 (22)	0.346	0.605
Any respiratory support — no./total no. (%)	89/186 (48)	84/178 (47)	0.229	0.535
Mechanical ventilation — no./total no. (%)	27/186 (15)	36/178 (20)	0.761	0.783

[a] Nine participants with both RSV subgroups A and B identified were excluded from this table. Percentages may not total 100 due to rounding. For demographic features, two-tailed Mann–Whitney U tests were used to compare continuous variables between the two groups; two-sided chi-square tests with Yates' correction or two-sided Fisher's exact tests were used to compare categorical variables between the two groups, whichever is appropriate.

[b] Two-sided multiple linear regression was used to adjust for the duration between symptom onset and sampling.

[c] Multiple linear regression, ordered logistic regression, or multivariable logistic regression (all two-sided) was used to adjust for covariates, depending on the type of the response (dependent) variable. Covariates included age, gestational age, sex, comorbidity, sampling season and country, study cohort, and peak RSV read count along with the duration between symptom onset and sampling. Models with different combinations of the covariates were tested, and the model with the lowest Akaike information criterion (AIC) was selected.

Abbreviations: *IQR* interquartile range, *PICU* paediatric intensive care unit, *RSV* respiratory syncytial virus, *SD* standard deviation.

Supplementary Table 5 | Characteristics of the RSV-infected infants with and without co-detection of any other virus (N = 418).[a]

	Presence of any other virus (N = 111)	Absence of any other virus (N = 307)	P value	Q value
**Demographic features**				
Age				
Median (IQR) — mo	4.6 (2.4–8.0)	3.8 (1.7–7.0)	0.028	
Distribution — no./total no. (%)			0.160	
<3 mo	35/110 (32)	129/306 (42)		
3 to <6 mo	35/110 (32)	80/306 (26)		
6 to <12 mo	40/110 (36)	97/306 (32)		
Gestational age				
Median (IQR) — wk	39.7 (38.4–40.6)	39.6 (38.7–40.4)	0.956	
Distribution — no./total no. (%)			0.181	
<32 wk	3/109 (3)	5/303 (2)		
32 to <37 wk	7/109 (6)	9/303 (3)		
≥37 wk	99/109 (91)	289/303 (95)		
Female sex — no./total no. (%)	45/110 (41)	137/306 (45)	0.556	
Comorbidity — no./total no. (%)	14/110 (13)	21/307 (7)	0.087	
**Virological features**				
RSV-A — no./total no. (%)[b]	56/108 (52)	152/301 (50)	0.910	
Peak RSV read count — total no.	108	299		
Mean ± SD — $\log_{10}$	3.8 ± 1.1	4.1 ± 1.0	0.024[c]	
**Clinical features**[d]				
ReSVinet score				
Mean ± SD	7.7 ± 5.1	7.6 ± 4.5	0.094	0.132
Distribution — no./total no. (%)			0.024	0.057
0–7	58/109 (53)	171/296 (58)		
8–13	35/109 (32)	84/296 (28)		
14–20	16/109 (15)	41/296 (14)		
Fever — no./total no. (%)	36/109 (33)	101/296 (34)	0.276	0.322
Hospitalisation — no./total no. (%)	53/99 (54)	159/293 (54)	0.464	0.464
PICU admission — no./total no. (%)	25/99 (25)	49/293 (17)	6.8×10^{-4}	0.005
Any respiratory support — no./total no. (%)	46/89 (52)	128/271 (47)	0.061	0.107
Mechanical ventilation — no./total no. (%)	21/89 (24)	43/271 (16)	0.003	0.010

[a] Fifteen patients with an equivocal presence of any other virus were removed from this table. For demographic features, two-tailed Mann–Whitney U tests were used to compare continuous variables between the two groups, and two-sided chi-square tests with Yates' correction or two-sided Fisher's exact tests were used to compare categorical variables between the two groups, whichever is appropriate.

[b] Nine participants with both RSV subgroups A and B identified were excluded from this comparison. Two-sided multivariable logistic regression was used to adjust for sampling season.

[c] Two-sided multiple linear regression was used to adjust for the duration between symptom onset and sampling.

[d] Multiple linear regression, ordered logistic regression, or multivariable logistic regression (all two-sided) was used to adjust for covariates, depending on the type of the response (dependent) variable. Covariates included age, gestational age, sex, comorbidity, sampling season and country, study cohort, RSV subgroup, and peak

RSV read count along with the duration between symptom onset and sampling. Models with different combinations of the covariates were tested, and the model with the lowest Akaike information criterion (AIC) was selected.
Abbreviations: *IQR* interquartile range, *PICU* paediatric intensive care unit, *RSV* respiratory syncytial virus, *SD* standard deviation.

Supplementary Table 6 | The presence and absence of co-detected viruses in samples from RSV-infected infants (N = 433).[a]

	Presence	Absence	Equivocal
Any non-RSV virus	111 (26)	307 (71)	15 (3)
Enterovirus (any)	74 (17)	349 (81)	10 (2)
– Rhinovirus	68 (16)	357 (82)	8 (2)
– Enterovirus D	3 (1)	429 (99)	1 (<1)
– Enterovirus A	2 (<1)	429 (99)	2 (<1)
– Enterovirus B	1 (<1)	431 (>99)	1 (<1)
Human coronavirus (any)[b]	15 (3)	416 (96)	2 (<1)
– HKU1	6 (1)	427 (99)	0
– OC43	6 (1)	426 (98)	1 (<1)
– NL63	2 (<1)	430 (99)	1 (<1)
– 229E	1 (<1)	432 (>99)	0
Human adenovirus	10 (2)	422 (97)	1 (<1)
Human herpesvirus 6	7 (2)	426 (98)	0
Human bocavirus	5 (1)	428 (99)	0
Human parechovirus A	4 (1)	425 (98)	4 (1)
Human cytomegalovirus	2 (<1)	429 (99)	2 (<1)
Human parainfluenza virus	2 (<1)	429 (99)	2 (<1)
Influenza C virus	1 (<1)	432 (>99)	0

[a] Data are shown as number (percentage). Percentages may not total 100 due to rounding.
[b] Some samples were collected in late 2019 and early 2020 (after the emergence of SARS-CoV-2).

Supplementary Table 7 | Characteristics of the RSV-infected infants with and without co-detection of enterovirus (N = 423).[a]

	Presence of enterovirus (N = 74)	Absence of enterovirus (N = 349)	P value
**Demographic features**			
Age			
Median (IQR) — mo	4.1 (2.0–6.7)	4.1 (1.8–7.5)	0.622
Distribution — no./total no. (%)			0.353
<3 mo	27/73 (37)	138/348 (40)	
3 to <6 mo	25/73 (34)	91/348 (26)	
6 to <12 mo	21/73 (29)	119/348 (34)	
Gestational age			
Median (IQR) — wk	39.6 (38.0–40.3)	39.6 (38.7–40.4)	0.415
Distribution			0.143
<32 wk	3/72 (4)	5/345 (1)	
32 to <37 wk	4/72 (6)	12/345 (3)	
≥37 wk	65/72 (90)	328/345 (95)	
Female sex — no./total no. (%)	27/73 (37)	158/348 (45)	0.235
Comorbidity — no./total no. (%)	8/73 (11)	28/349 (8)	0.558
**Virological features**			
RSV-A — no./total no. (%)[b]	41/73 (56)	172/341 (50)	0.822
Peak RSV read count — total no.	71	341	
Mean ± SD — $\log_{10}$	3.8 ± 1.0	4.1 ± 1.0	0.057[c]
**Clinical features**[d]			
ReSVinet score			
Mean ± SD	7.6 ± 5.1	7.6 ± 4.6	0.082
Distribution — no./total no. (%)			0.072
0–7	39/72 (54)	195/338 (58)	
8–13	23/72 (32)	95/338 (28)	
14–20	10/72 (14)	48/338 (14)	
Fever — no./total no. (%)	23/72 (32)	116/338 (34)	0.722
Hospitalisation — no./total no. (%)	35/64 (55)	179/334 (54)	0.258
PICU admission — no./total no. (%)	14/64 (22)	59/334 (18)	0.106
Any respiratory support — no./total no. (%)	30/59 (51)	145/307 (47)	0.301
Mechanical ventilation — no./total no. (%)	12/59 (20)	52/307 (17)	0.408

[a] Ten infants with an equivocal presence of any enterovirus were removed from this table. Percentages may not total 100 due to rounding. For demographic features, two-tailed Mann–Whitney U tests were used to compare continuous variables between the two groups, and two-sided chi-square tests with Yates' correction or two-sided Fisher's exact tests were used to compare categorical variables between the two groups, whichever is appropriate.

[b] Nine participants with both RSV subgroups A and B identified were excluded from this comparison. Two-sided multivariable logistic regression was used to adjust for sampling season.

[c] Two-sided multiple linear regression was used to adjust for the duration between symptom onset and sampling.

[d] Multiple linear regression, ordered logistic regression, or multivariable logistic regression (all two-sided) was used to adjust for covariates, depending on the type of the response (dependent) variable. Covariates included age, gestational age, sex, comorbidity, sampling season and country, study cohort, RSV subgroup, and peak

RSV read count along with the duration between symptom onset and sampling. Models with different combinations of the covariates were tested, and the model with the lowest Akaike information criterion (AIC) was selected.
Abbreviations: *IQR* interquartile range, *PICU* paediatric intensive care unit, *RSV* respiratory syncytial virus, *SD* standard deviation.

Supplementary Table 8 | Characteristics of the RSV-infected infants with and without co-detection of seasonal coronaviruses (N = 431).[a]

	Presence of coronavirus (N = 15)	Absence of coronavirus (N = 416)	P value
**Demographic features**			
Age			
Median (IQR) — mo	8.2 (4.2–10.0)	4.0 (1.8–7.3)	0.008
Distribution — no./total no. (%)			0.101
<3 mo	3/15 (20)	165/414 (40)	
3 to <6 mo	3/15 (20)	114/414 (28)	
6 to <12 mo	9/15 (60)	135/414 (33)	
Gestational age			
Median (IQR) — wk	40.1 (39.7–40.9)	39.6 (38.4–40.4)	0.076
Distribution			0.604
<32 wk	0/15 (0)	8/410 (2)	
32 to <37 wk	1/15 (7)	16/410 (4)	
≥37 wk	14/15 (93)	386/410 (94)	
Female sex — no./total no. (%)	8/15 (53)	183/414 (44)	0.664
Comorbidity — no./total no. (%)	2/15 (13)	35/415 (8)	0.376
**Virological features**			
RSV-A — no./total no. (%)[b]	7/15 (47)	211/407 (52)	0.881
Peak RSV read count — total no.	15	405	
Mean ± SD — $\log_{10}$	4.4 ± 0.6	4.0 ± 1.1	0.228[c]
**Clinical features**[d]			
ReSVinet score			
Mean ± SD	7.1 ± 4.2	7.6 ± 4.6	0.419
Distribution — no./total no. (%)			0.318
0–7	10/15 (67)	228/403 (57)	
8–13	4/15 (27)	118/403 (29)	
14–20	1/15 (7)	57/403 (14)	
Fever — no./total no. (%)	8/15 (53)	134/403 (33)	0.973
Hospitalisation — no./total no. (%)	5/13 (38)	213/389 (55)	0.322
PICU admission — no./total no. (%)	3/13 (23)	74/389 (19)	0.0499[e]
Any respiratory support — no./total no. (%)	4/12 (33)	175/358 (49)	0.287
Mechanical ventilation — no./total no. (%)	2/12 (17)	64/358 (18)	0.232

[a] Two infants with an equivocal presence of any coronavirus were removed from this table. Percentages may not total 100 due to rounding. For demographic features, two-tailed Mann–Whitney U tests were used to compare continuous variables between the two groups, and two-sided chi-square tests with Yates' correction or two-sided Fisher's exact tests were used to compare categorical variables between the two groups, whichever is appropriate.

[b] Nine participants with both RSV subgroups A and B identified were excluded from this comparison. Two-sided multivariable logistic regression was used to adjust for sampling season.

[c] Two-sided multiple linear regression was used to adjust for the duration between symptom onset and sampling.

[d] Multiple linear regression, ordered logistic regression, or multivariable logistic regression (all two-sided) was used to adjust for covariates, depending on the type of the response (dependent) variable. Covariates included age, gestational age, sex, comorbidity, sampling season and country, study cohort, RSV subgroup, and peak

RSV read count along with the duration between symptom onset and sampling. Models with different combinations of the covariates were tested, and the model with the lowest Akaike information criterion (AIC) was selected.

[e] After adjusting for multiple comparisons, the Q value is 0.349.

Abbreviations: *IQR* interquartile range, *PICU* paediatric intensive care unit, *RSV* respiratory syncytial virus, *SD* standard deviation.

Supplementary Table 9 | Characteristics of the RSV-infected infants with and without co-detection of human adenovirus (N = 432).[a]

	Presence of human adenovirus (N = 10)	Absence of human adenovirus (N = 422)	P value
**Demographic features**			
Age			
Median (IQR) — mo	4.5 (4.1–8.5)	4.1 (1.8–7.4)	0.365
Distribution — no./total no. (%)			0.403
<3 mo	2/10 (20)	167/420 (40)	
3 to <6 mo	4/10 (40)	113/420 (27)	
6 to <12 mo	4/10 (40)	140/420 (33)	
Gestational age			
Median (IQR) — wk	39.4 (38.3–40.3)	39.6 (38.6–40.4)	0.772
Distribution			0.457
<32 wk	0/10 (0)	8/416 (2)	
32 to <37 wk	1/10 (10)	16/416 (4)	
≥37 wk	9/10 (90)	392/416 (94)	
Female sex — no./total no. (%)	5/10 (50)	186/420 (44)	0.756
Comorbidity — no./total no. (%)	1/10 (10)	36/421 (9)	0.597
**Virological features**			
RSV-A — no./total no. (%)[b]	6/10 (60)	213/413 (52)	0.489
Peak RSV read count — total no.	10	411	
Mean ± SD — $\log_{10}$	3.4 ± 1.5	4.0 ± 1.0	0.136[c]
**Clinical features**[d]			
ReSVinet score			
Mean ± SD	5.9 ± 4.9	7.6 ± 4.6	0.044[e]
Distribution — no./total no. (%)			0.561
0–7	6/10 (60)	233/409 (57)	
8–13	3/10 (30)	119/409 (29)	
14–20	1/10 (10)	57/409 (14)	
Fever — no./total no. (%)	4/10 (40)	137/409 (34)	0.959
Hospitalisation — no./total no. (%)	4/10 (40)	215/393 (55)	0.049[e]
PICU admission — no./total no. (%)	1/10 (10)	76/393 (19)	0.635
Any respiratory support — no./total no. (%)	4/7 (57)	175/364 (48)	0.933
Mechanical ventilation — no./total no. (%)	1/7 (14)	65/364 (18)	0.577

[a] One infant with an equivocal presence of human adenovirus were removed from this table. For demographic features, two-tailed Mann–Whitney U tests were used to compare continuous variables between the two groups, and two-sided Fisher's exact tests were used to compare categorical variables between the two groups.
[b] Nine participants with both RSV subgroups A and B identified were excluded from this comparison. Two-sided multivariable logistic regression was used to adjust for sampling season.
[c] Two-sided multiple linear regression was used to adjust for the duration between symptom onset and sampling.
[d] Multiple linear regression, ordered logistic regression, or multivariable logistic regression (all two-sided) was used to adjust for covariates, depending on the type of the response (dependent) variable. Covariates included age, gestational age, sex, comorbidity, sampling season and country, study cohort, RSV subgroup, and peak RSV read count along with the duration between symptom onset and sampling. Models with different

combinations of the covariates were tested, and the model with the lowest Akaike information criterion (AIC) was selected.

[e] After adjusting for multiple comparisons, the Q value is 0.173.

Abbreviations: *IQR* interquartile range, *PICU* paediatric intensive care unit, *RSV* respiratory syncytial virus, *SD* standard deviation.

Supplementary Table 10 | Characteristics of the RSV-infected infants with and without co-detection of human herpesvirus 6 (HHV-6) (N = 433).[a]

	Presence of HHV-6 (N = 7)	Absence of HHV-6 (N = 426)	P value
**Demographic features**			
Age			
Median (IQR) — mo	1.6 (1.2–4.5)	4.1 (1.9–7.5)	0.137
Distribution — no./total no. (%)			0.159
<3 mo	5/7 (71)	164/424 (39)	
3 to <6 mo	0/7 (0)	117/424 (28)	
6 to <12 mo	2/7 (29)	143/424 (34)	
Gestational age			
Median (IQR) — wk	39.3 (38.3–40.2)	39.6 (38.6–40.4)	0.538
Distribution			0.347
<32 wk	0/7 (0)	8/420 (2)	
32 to <37 wk	1/7 (14)	16/420 (4)	
≥37 wk	6/7 (86)	396/420 (94)	
Female sex — no./total no. (%)	3/7 (43)	189/424 (45)	1.000
Comorbidity — no./total no. (%)	1/7 (14)	36/425 (8)	0.468
**Virological features**			
RSV-A — no./total no. (%)[b]	1/7 (14)	219/417 (53)	0.237
Peak RSV read count — total no.	7	415	
Mean ± SD — log_{10}	3.7 ± 1.5	4.0 ± 1.0	0.540[c]
**Clinical features**[d]			
ReSVinet score			
Mean ± SD	9.6 ± 4.4	7.5 ± 4.6	0.906
Distribution — no./total no. (%)			0.977
0–7	1/7 (14)	239/413 (58)	
8–13	5/7 (71)	117/413 (28)	
14–20	1/7 (14)	57/413 (14)	
Fever — no./total no. (%)	0/7 (0)	142/413 (34)	0.985
Hospitalisation — no./total no. (%)	6/7 (86)	213/397 (54)	0.882
PICU admission — no./total no. (%)	5/7 (71)	72/397 (18)	0.021[e]
Any respiratory support — no./total no. (%)	6/6 (100)	173/366 (47)	0.987
Mechanical ventilation — no./total no. (%)	3/6 (50)	63/366 (17)	0.311

[a] Percentages may not total 100 due to rounding. For demographic features, two-tailed Mann–Whitney U tests were used to compare continuous variables between the two groups, and two-sided Fisher's exact tests were used to compare categorical variables between the two groups.

[b] Nine participants with both RSV subgroups A and B identified were excluded from this comparison. Two-sided multivariable logistic regression was used to adjust for sampling season.

[c] Two-sided multiple linear regression was used to adjust for the duration between symptom onset and sampling.

[d] Multiple linear regression, ordered logistic regression, or multivariable logistic regression (all two-sided) was used to adjust for covariates, depending on the type of the response (dependent) variable. Covariates included age, gestational age, sex, comorbidity, sampling season and country, study cohort, RSV subgroup, and peak RSV read count along with the duration between symptom onset and sampling. Models with different combinations of the covariates were tested, and the model with the lowest Akaike information criterion (AIC) was selected.

[e] After adjusting for multiple comparisons, the Q value is 0.147.
Abbreviations: *HHV-6* human herpesvirus 6, *IQR* interquartile range, *PICU* paediatric intensive care unit, *RSV* respiratory syncytial virus, *SD* standard deviation.

Supplementary Table 11 | Phylogenetic signals between the RSV phylogenies and commonly co-detected pathogens.[a]

A. RSV-A (N = 207)

	RV	HCoV	HAdV	HHV-6
Presence (n)	36	7	5	1
D value	0.939	1.057	1.296	—
Probability of random distribution of the virus	0.285	0.608	0.772	—
Probability of distribution of the virus under Brownian motion	0	0	0.001	—

	M. catarrhalis	*S. pneumoniae*	*H. influenzae*	*S. aureus*
Presence (n)	146	118	91	19
D value	0.989	0.989	0.990	0.937
Probability of random distribution of the bacterium	0.433	0.430	0.441	0.342
Probability of distribution of the bacterium under Brownian motion	0	0	0	0

B. RSV-B (N = 177)

	RV	HCoV	HAdV	HHV-6
Presence (n)	23	8	2	4
D value	0.931	0.707	—	0.959
Probability of random distribution of the virus	0.357	0.198	—	0.517
Probability of distribution of the virus under Brownian motion	0	0.045	—	0.039

	M. catarrhalis	*S. pneumoniae*	*H. influenzae*	*S. aureus*
Presence (n)	114	85	60	15
D value	1.007	0.774	0.919	0.762
Probability of random distribution of the bacterium	0.515	0.022	0.240	0.156
Probability of distribution of the bacterium under Brownian motion	0	0	0	0.002

[a] Only samples with at least 70% of the RSV coding sequences recovered were included in this analysis. For infants with multiple samples collected, only the sample with the highest coverage was included. Viruses that were co-detected with no more than three RSV strains on the phylogeny were not tested for phylogenetic signal. Abbreviations: *HAdV* human adenovirus, *HCoV* human coronavirus, *HHV-6* human herpesvirus 6, *H. influenzae* Haemophilus influenzae, *M. catarrhalis* Moraxella catarrhalis, *RSV* respiratory syncytial virus, *RV* human rhinovirus, *S. aureus* Staphylococcus aureus, *S. pneumoniae* Streptococcus pneumoniae.

Supplementary Table 12 | The presence and absence of each identified bacterial genus in the RSV-infected infants (N = 433).[a]

	Presence	Absence	Equivocal
Moraxella	327 (76)	99 (23)	7 (2)
– *M. catarrhalis*	286 (66)	139 (32)	8 (2)
Streptococcus	296 (68)	77 (18)	60 (14)
– *S. pneumoniae*	222 (51)	188 (43)	23 (5)
Haemophilus	194 (45)	225 (52)	14 (3)
– *H. influenzae*	164 (38)	262 (61)	7 (2)
Neisseria	50 (12)	362 (84)	21 (5)
– *N. meningitidis*	2 (<1)	431 (>99)	0
Staphylococcus	49 (11)	373 (86)	11 (3)
– *S. aureus*	38 (9)	389 (90)	6 (1)
Burkholderia	37 (9)	362 (84)	34 (8)
Dolosigranulum[b]	34 (8)	382 (88)	17 (4)
Gemella[b]	19 (4)	400 (92)	14 (3)
Enterococcus[b]	16 (4)	406 (94)	11 (3)
Granulicatella[b]	8 (2)	417 (96)	8 (2)
Rothia[b]	7 (2)	422 (97)	4 (1)
Enterobacter	5 (1)	427 (99)	1 (<1)
Escherichia	5 (1)	427 (99)	1 (<1)
Corynebacterium[b]	5 (1)	419 (97)	9 (2)
Pasteurella[b]	3 (1)	425 (98)	5 (1)
Veillonella[b]	3 (1)	423 (98)	7 (2)
Klebsiella	2 (<1)	430 (99)	1 (<1)
Aggregatibacter[b]	2 (<1)	429 (99)	2 (<1)
Citrobacter[b]	1 (<1)	432 (>99)	0
Xanthomonas[b]	1 (<1)	432 (>99)	0
Lactococcus[b]	1 (<1)	431 (>99)	1 (<1)
Acinetobacter	1 (<1)	430 (99)	2 (<1)
Actinomyces[b]	1 (<1)	429 (99)	3 (1)
Stenotrophomonas	1 (<1)	429 (99)	3 (1)
Pseudomonas	1 (<1)	422 (97)	10 (2)

[a] Data are shown as number (percentage). Percentages may not total 100 due to rounding.

[b] These genera were not included in the *Castanet* enrichment panel, so their read numbers were expected to be lower as these were detected by unenriched metagenomics only. With *Castanet*, as with all capture-based protocols, targeted sequences are selectively amplified by 100–1000x during library preparation, but untargeted sequences remain at a lower level (commonly referred to as "metagenomic background" in this context), and are subsequently sequenced alongside the targeted ones. We used the PubMLST multi-species isolate database to identify matching bacteria, thus allowing us to classify contig sequences (assembled from the sequencing reads) to a certain taxonomic level, even though the genera and species were not part of the *Castanet* panel.

Supplementary Table 13 | Characteristics of the RSV-infected infants by breastfeeding status within the 4 weeks prior to the RSV infection (N = 51).[a]

	Breastfed[b] (N = 27)	Not breastfed (N = 24)	Unadj. P value	Adj. P value
**Demographic features**				
Age				
Median (IQR) — mo	5.6 (3.3–8.9)	8.1 (3.7–10.4)	0.282	
Distribution — no. (%)			0.805	
<3 mo	6 (22)	4 (17)		
3 to <6 mo	8 (30)	6 (25)		
6 to <12 mo	13 (48)	14 (58)		
Gestational age				
Median (interquartile range) — wk	39.7 (38.9–41.2)	39.4 (38.7–40.3)	0.230	
Female sex — no. (%)	12 (44)	13 (54)	0.680	
**Virological features**				
RSV-A — no. (%)	7 (26)	10 (42)	0.538[c]	
Peak RSV read count — total no.	26	23		
Mean ± SD — $\log_{10}$	3.8 ± 1.3	4.1 ± 1.1	0.152[d]	
**Clinical features**[e]				
ReSVinet score				
Mean ± SD	4.5 ± 2.1	5.9 ± 2.9	0.078	0.103
Distribution — no./total no. (%)			0.285	0.239
0–7	24/26 (92)	18/23 (78)		
8–13	2/26 (8)	4/23 (17)		
14–20	0/26 (0)	1/23 (4)		
Fever — no./total no. (%)	10/26 (38)	14/23 (61)	0.201	0.241
Hospitalisation — no./total no. (%)	1/24 (4)	1/18 (6)	1.000	0.999
PICU admission — no./total no. (%)	0/24 (0)	1/18 (6)	0.429	1.000
Any respiratory support — no./total no. (%)	0/24 (0)	1/18 (6)	0.429	1.000
Mechanical ventilation — no./total no. (%)	0/20 (0)	0/18 (0)	NA	NA

[a] All 51 infants were born ≥37 weeks' gestation and had no comorbidity. Percentages may not total 100 due to rounding. For demographic features, two-tailed Mann–Whitney U tests were used to compare continuous variables between the two groups; two-sided chi-square tests with Yates' correction or two-sided Fisher's exact tests were used to compare categorical variables between the two groups, whichever is appropriate.

[b] Breastfed was defined as either exclusively breastfed or in combination with formula milk.

[c] Two-sided multivariable logistic regression was used to adjust for sampling season.

[d] Two-sided multiple linear regression was used to adjust for the duration between symptom onset and sampling.

[e] Multiple linear regression, ordered logistic regression, or multivariable logistic regression (all two-sided) was used to determine the unadjusted and adjusted P values, depending on the type of the response (dependent) variable. Covariates included age, gestational age, sex, sampling season and country, RSV subgroup, peak RSV read count, and the duration between symptom onset and sampling. Models with different combinations of the covariates were tested, and the model with the lowest Akaike information criterion (AIC) was selected.

Abbreviations: *Adj.* adjusted, *IQR* interquartile range, *PICU* paediatric intensive care unit, *RSV* respiratory syncytial virus, *SD* standard deviation, *Unadj.* unadjusted.

Supplementary Table 14 | Frequency of known ribosomal sequence types of the co-detected bacteria and the most likely corresponding sequence type and serotype combination.

Frequency	Ribosomal sequence type	Sequence type[a]	Clonal complex[a]	Serotype[b]	%[c]	Total # of isolates in PubMLST[d]
Streptococcus pneumoniae						
1	614	138	-	6B	75	73
2	644	62	-	11A	59	145
2	12187	2062	-	19A	84	90
1	12227	66	-	9N	73	60
1	12688	30	-	Inconclusive	80	10
1	13052	1925	-	19A	100	4
1	13435	439	-	23B	56	155
2	13906	1262	-	15BC	62	166
1	21764	392	-	17F	56	9
1	22747	1349	-	Genetic variant	42	12
1	22892	4149	-	Inconclusive	81	16
1	102491	2105	-	15A	38	8
2	104663	3811	-	15A	100	12
1	151444	452	-	Inconclusive	100	8
1	151477	2669	-	Inconclusive	88	8
1	151481	179	-	Inconclusive	100	3
Haemophilus influenzae						
1	24137	1877	ST-584	NT	31	16
2	24162	12	ST-12	NT	66	44
1	24179	422	ST-422	NT	58	31
1	24185	165	ST-165	NT	76	25
1	24208	57	ST-57	NT	78	18
2	49634	105	ST-105	NT	100	1
1	66853	368	ST-1836	NT	88	8
1	66856	159	ST-107	NT	89	18
1	66889	145	ST-11	NT	88	16
1	72352	266	ST-266	NT	70	10
2	89110	160	ST-487	NT	84	19
1	89135	567	ST-746	NT	75	4
1	89141	1218	ST-107	NT	93	14
1	91528	932	-	NT	100	1
1	98191	836	ST-836	NT	70	10
1	125128	2092/2597	ST-1025	ND/NT	50	2
1	125166	597	ST-584	NT	75	4
1	125211	3	ST-3	NT	100	2
1	126121	1238	ST-931	NT	100	1
1	126127	-	-	-	-	-
1	132982	472	ST-472	NT	60	5
2	133371	-	-	-	-	-
Escherichia coli						
1	1674	1193/53	ST-14	-	60	5
1	2135	69/3	ST-69	UPEC	54	94
1	93310	-	-	-	-	-

Frequency	Ribosomal sequence type	Sequence type[a]	Clonal complex[a]	Serotype[b]	%[c]	Total # of isolates in PubMLST[d]
Neisseria meningitidis						
1	2327	11	ST-11	W	87	2279
Moraxella catarrhalis						
1	49012	-	-	-	-	-
1	89989	-	-	-	-	-
1	92879	-	-	-	-	-
1	92972	-	-	-	-	-
2	105842	-	-	-	-	-
1	131792	-	-	-	-	-
1	131807	-	-	-	-	-

[a] Sequence type and clonal complex are based on the multi-locus sequence typing (MLST) scheme. For *E. coli*, sequence type based on the Achtman MLST scheme is shown first, followed by sequence type based on the Pasteur MLST scheme.

[b] For *E. coli*, phenotype is shown in the serotype column. For *N. meningitidis*, capsule group is shown in the serotype column.

[c] Percentage of this most frequent combination of the ribosomal sequence type, sequence type, clonal complex, and serotype.

[d] Total number of isolates with this specified ribosomal sequence type found in PubMLST (https://pubmlst.org/organisms) as of 1st December 2022.

Abbreviations: *ND* not determined; *NT* nontypeable, *UPEC* uropathogenic E. coli.

Supplementary Table 15 | Characteristics of the RSV-infected infants with and without co-detection of any *Haemophilus* species (N = 419).[a]

	Presence of any *Haemophilus* sp. (N = 194)	Absence of any *Haemophilus* sp. (N = 225)	P value	Q value	Cohen's f^2 [b]
**Demographic features**					
Age					
Median (IQR) — mo	5.2 (2.0–8.7)	3.5 (1.7–5.9)	1.6×10^{-4}		
Distribution			4.2×10^{-6}		
<3 mo	70/194 (36)	93/223 (42)			
3 to <6 mo	37/194 (19)	78/223 (35)			
6 to <12 mo	87/194 (45)	52/223 (23)			
Gestational age					
Median (IQR) — wk	39.6 (38.3–40.3)	39.7 (38.7–40.7)	0.107		
Distribution			0.368		
<32 wk	5/192 (3)	2/221 (1)			
32 to <37 wk	9/192 (5)	8/221 (4)			
≥37 wk	178/192 (93)	211/221 (95)			
Female sex	84/194 (43)	101/223 (45)	0.757		
Comorbidity	20/194 (10)	16/224 (7)	0.329		
**Virological features**					
RSV-A[c]	109/192 (57)	103/218 (47)	0.009		
Peak RSV read count	n = 192	n = 216			
Mean ± SD — $\log_{10}$	4.0 ± 0.9	4.1 ± 1.1	0.745[d]		
**Clinical features**[e]					
ReSVinet score					
Mean ± SD	8.5 ± 4.5	6.8 ± 4.6	4.4×10^{-6}	3.1×10^{-5}	0.055
Distribution			7.8×10^{-4}	1.8×10^{-3}	0.020
0–7	92/191 (48)	140/215 (65)			
8–13	68/191 (36)	49/215 (23)			
14–20	31/191 (16)	26/215 (12)			
Fever	85/191 (44)	54/215 (25)	0.020	0.023	0.013
Hospitalisation	112/178 (63)	99/213 (46)	1.9×10^{-4}	6.5×10^{-4}	0.066
PICU admission	43/178 (24)	33/213 (15)	0.118	0.118	0.010
Any respiratory support	90/164 (55)	83/196 (42)	0.019	0.023	0.025
Mechanical ventilation	40/164 (24)	25/196 (13)	0.019	0.023	0.031

[a] Fourteen patients with an equivocal presence of any *Haemophilus* sp. were excluded from the table. *Haemophilus* spp. included *H. influenzae* (80%), *H. haemolyticus* (10%), *H. parainfluenzae* (7%), *H. parahaemolyticus* (1%) and unclassified species (1%). Unless otherwise specified, data are shown as number/total number (%). Percentages may not total 100 due to rounding. For demographic features, two-tailed Mann–Whitney U tests were used to compare continuous variables between the two groups, and two-sided chi-square tests with Yates' correction or two-sided Fisher's exact tests were used to compare categorical variables between the two groups, whichever is appropriate.

[b] Cohen's f^2 was used to evaluate the effect size of the presence of any *Haemophilus* sp. on different clinical features. A value between 0.02 and <0.15 represents a small effect size.

[c] Nine participants with both RSV subgroups A and B identified were excluded from this comparison. Two-sided multivariable logistic regression was used to adjust for sampling season.

[d] Two-sided multiple linear regression was used to adjust for the duration between symptom onset and sampling.
[e] Multiple linear regression, ordered logistic regression, or multivariable logistic regression (all two-sided) was used to adjust for covariates, depending on the type of the response (dependent) variable. Covariates included age, gestational age, sex, comorbidity, sampling season and country, study cohort, RSV subgroup, and peak RSV read count along with the duration between symptom onset and sampling. Models with different combinations of the covariates were tested, and the model with the lowest Akaike information criterion (AIC) was selected.
Abbreviations: *IQR* interquartile range, *PICU* paediatric intensive care unit, *RSV* respiratory syncytial virus, *SD* standard deviation.

Supplementary Table 16 | Documented clinical isolates from either bronchial wash or a nasopharyngeal swab (N = 27).[a]

Clinical bacterial isolates	Frequency	Recovery rate by targeted metagenomics
Staphylococcus aureus	17	65%
Haemophilus influenzae	9	100%
Moraxella or *M. catarrhalis*	9	67%
Streptococcus pneumoniae	6	83%
Klebsiella pneumoniae	2	0%
Escherichia coli	1	0%
Streptococcus pyogenes	1	0%
Streptococcus agalactiae	1	100%
Enterobacter cloacae complex	1	100%

[a] Among the 433 infants, 30 had documented bacterial isolates clinically, including four from blood samples, 27 from bronchial wash samples, one from a nasopharyngeal swab, and one without available information about the sample type (two infants had bacteria isolated from both blood and bronchial wash, and one infant had bacteria isolated from both bronchial wash and the nasopharyngeal swab). All infants with documented bacterial isolates received antibiotics. Information on the timing of sampling of these clinical samples was not available.

Supplementary Table 17 | Characteristics of the infants with co-detection of RSV and any *Haemophilus* species who received or did not receive antibiotics during the infection (N = 163).[a]

	Received antibiotics (N = 53)	Not received antibiotics (N = 110)	P value	Q value
**Demographic features**				
Age				
Median (IQR) — mo	2.1 (1.0–6.2)	6.0 (2.8–8.9)	8.4×10^{-5}	
Distribution — no. (%)			0.002	
<3 mo	31 (58)	33 (30)		
3 to <6 mo	8 (15)	22 (20)		
6 to <12 mo	14 (26)	55 (50)		
Gestational age				
Median (IQR) — wk	38.7 (37.1–39.6)	39.9 (38.8–40.4)	8.4×10^{-5}	
Distribution — no./total no. (%)			0.022	
<32 wk	3/53 (6)	2/108 (2)		
32 to <37 wk	6/53 (11)	3/108 (3)		
≥37 wk	44/53 (83)	103/108 (95)		
Female sex — no. (%)	19 (36)	48 (44)	0.437	
Comorbidity — no. (%)	12 (23)	8 (7)	0.011	
**Virological features**				
RSV-A — no./total no. (%)[b]	23/52 (44)	65/109 (60)	0.555	
Peak RSV read count				
Mean ± SD — $\log_{10}$	4.0 ± 0.8	4.0 ± 1.0	0.532[c]	
**Clinical features**[d]				
ReSVinet score				
Mean ± SD	13.1 ± 3.9	7.1 ± 3.5	2.0×10^{-11}	1.4×10^{-10}
Distribution — no./total no. (%)			1.2×10^{-5}	2.8×10^{-5}
0–7	6/53 (11)	61/109 (56)		
8–13	22/53 (42)	42/109 (39)		
14–20	25/53 (47)	6/109 (6)		
Fever — no./total no. (%)	20/53 (38)	52/109 (48)	0.559	0.559
Hospitalisation — no. (%)	49 (92)	63 (57)	0.002	0.003
PICU admission — no. (%)	35 (66)	8 (7)	6.6×10^{-6}	2.3×10^{-5}
Any respiratory support — no./total no. (%)	47/53 (89)	43/96 (45)	0.004	0.004
Mechanical ventilation — no./total no. (%)	34/53 (64)	6/96 (6)	2.9×10^{-4}	5.2×10^{-4}

[a] One hundred and ninety-four RSV-infected infants had co-detection of any *Haemophilus* species. Among them, 31 had no available information on antibiotic use and were excluded from the table. Percentages may not total 100 due to rounding. For demographic features, two-tailed Mann–Whitney U tests were used to compare continuous variables between the two groups, and two-sided chi-square tests with Yates' correction or two-sided Fisher's exact tests were used to compare categorical variables between the two groups, whichever is appropriate.

[b] Two participants with both RSV subgroups A and B identified were excluded from this comparison. Two-sided multivariable logistic regression was used to adjust for sampling season.

[c] Two-sided multiple linear regression was used to adjust for the duration between symptom onset and sampling.

[d] Multiple linear regression, ordered logistic regression, or multivariable logistic regression (all two-sided) was used to adjust for covariates, depending on the type of the response (dependent) variable. Covariates included age, gestational age, sex, comorbidity, sampling season and country, study cohort, RSV subgroup, and peak RSV read count along with the duration between symptom onset and sampling. Models with different combinations of the covariates were tested, and the model with the lowest Akaike information criterion (AIC) was selected.
Abbreviations: *IQR* interquartile range, *PICU* paediatric intensive care unit, *RSV* respiratory syncytial virus, *SD* standard deviation.

Supplementary Table 18 | Targeted bacterial and viral species.

**Bacteria**			
**Genus**	**Species**	**Genus**	**Species**
Acinetobacter	*baumannii*	*Leptospira*	spp.
	calcoaceticus	*Listeria*	*monocytogenes*
Bartonella	*henselae*	*Moraxella*	*catarrhalis*
Bordetella	*pertussis*	*Mycobacterium*	*avium*
Borreliella	*burgdorferi*		*intracellulare*
Brucella	spp.		*tuberculosis*
Burkholderia	*cepacia*	*Mycoplasmoides*	*pneumoniae*
Chlamydia	*pneumoniae*	*Neisseria*	*meningitidis*
	psittaci	*Nocardia*	spp.
Coxiella	*burnetii*	*Pseudomonas*	*aeruginosa*
Enterobacter	*cloacae*	*Serratia*	*marcescens*
Escherichia	*coli*	*Staphylococcus*	*aureus*
Haemophilus	*influenzae*	*Stenotrophomonas*	*maltophilia*
	parainfluenzae	*Streptococcus*	*agalactiae*
Klebsiella	*aerogenes*		*pneumoniae*
	pneumoniae		*pyogenes*
	oxytoca	*Treponema*	*pallidum*
Legionella	*pneumophila*		

**Viruses**	
**Family / Virus Name**	**Family / Virus Name**
Adenoviridae	*Orthomyxoviridae*
Human adenovirus	Influenza A virus
Arenaviridae	Influenza B virus
Lassa virus	Influenza C virus
Lymphocytic choriomeningitis virus	*Paramyxoviridae*
Coronaviridae	Hendra virus
Human coronavirus HKU1, NL63, OC43, 229E	Human parainfluenza virus 1, 2, 3, 4a, 4b
Middle East respiratory syndrome-related coronavirus	Measles virus
Severe acute respiratory syndrome coronavirus	Mumps virus
Flaviviridae	Nipah virus
Dengue virus	Parainfluenza virus 5
Japanese encephalitis virus	Sosuga virus
Murray Valley encephalitis virus	*Parvoviridae*
St. Louis encephalitis virus	Human bocavirus
Tick-borne encephalitis virus	Human parvovirus 4
West Nile virus	Human parvovirus B19
Yellow fever virus	*Peribunyaviridae*
Zika virus	California encephalitis virus
Matonaviridae	*Phenuiviridae*
Rubella virus	Rift valley fever virus
	Sandfly fever Naples virus
	Sandfly fever Sicilian virus

Family / Virus Name	Family / Virus Name
Orthoherpesviridae	
Human alphaherpesvirus 1, 2 (Herpes simplex virus type 1, 2)	
Human alphaherpesvirus 3 (Varicella-zoster virus)	
Human betaherpesvirus 5 (Human cytomegalovirus)	
Human betaherpesvirus 6A, 6B, 7 (Human herpesvirus 6A, 6B, 7)	
Human gammaherpesvirus 4 (Epstein-Barr virus)	
Human gammaherpesvirus 8 (Kaposi's sarcoma-associated herpesvirus)	
Picornaviridae	*Rhabdoviridae*
Cardiovirus A, B	Australian bat lyssavirus
Coxsackievirus A	Duvenhage lyssavirus
Echovirus	European bat lyssavirus 1, 2
Enterovirus A, B, D	Lagos bat lyssavirus
Hepatovirus A1 (Hepatitis A virus)	Mokola virus
Human parechovirus	Rabies lyssavirus
Parechovirus B1 (Ljunganvirus 1)	*Sedoreoviridae*
Rhinovirus A, B, C	Rotavirus A, B, C
Rosavirus A	*Togaviridae*
Salivirus	Chikungunya virus
Pneumoviridae	Eastern equine encephalitis virus
Human metapneumovirus	Venezuelan equine encephalitis virus
Human respiratory syncytial virus	Western equine encephalitis virus
Polyomaviridae	
BK polyomavirus	
JC polyomavirus	

[a] The probe set only targeted partial genomes of the members of the family *Orthoherpesviridae*.

Supplementary Table 19 | List of the GenBank accession numbers for RSV consensus sequences used in phylogenetic analyses.

RSV-A (N = 207)			RSV-B (N = 177)		
LR699315	MZ515752	MZ515957	LR699726	MZ515711	MZ515889
LR699734	MZ515766	MZ515958	LR699735	MZ515712	MZ515895
LR699736	MZ515773	MZ515959	LR699738	MZ515714	MZ515903
LR699737	MZ515777	MZ515960	LR699739	MZ515715	MZ515904
MZ515551	MZ515780	MZ515961	LR699740	MZ515719	MZ515918
MZ515555	MZ515782	MZ515962	LR699741	MZ515724	MZ515924
MZ515556	MZ515784	MZ515963	LR699742	MZ515725	MZ515925
MZ515559	MZ515788	MZ515966	LR699743	MZ515727	MZ515926
MZ515566	MZ515789	MZ515967	LR699744	MZ515728	MZ515930
MZ515567	MZ515790	MZ515968	MZ515553	MZ515732	MZ515938
MZ515568	MZ515800	MZ515969	MZ515554	MZ515733	MZ515944
MZ515569	MZ515801	MZ515971	MZ515557	MZ515738	MZ515946
MZ515570	MZ515802	MZ515983	MZ515558	MZ515743	MZ515947
MZ515571	MZ515803	MZ515984	MZ515560	MZ515745	MZ515950
MZ515572	MZ515821	MZ515985	MZ515562	MZ515747	MZ515953
MZ515573	MZ515825	MZ515987	MZ515563	MZ515748	MZ515955
MZ515575	MZ515828	MZ515992	MZ515565	MZ515751	MZ515970
MZ515577	MZ515833	MZ515993	MZ515574	MZ515756	MZ515972
MZ515582	MZ515834	MZ515994	MZ515578	MZ515761	MZ515975
MZ515583	MZ515835	MZ516000	MZ515581	MZ515762	MZ515991
MZ515592	MZ515840	MZ516002	MZ515584	MZ515765	MZ515997
MZ515597	MZ515841	MZ516005	MZ515586	MZ515769	MZ516003
MZ515604	MZ515842	MZ516008	MZ515590	MZ515770	MZ516006
MZ515606	MZ515848	MZ516011	MZ515591	MZ515771	MZ516016
MZ515609	MZ515850	MZ516012	MZ515595	MZ515775	MZ516020
MZ515614	MZ515851	MZ516014	MZ515598	MZ515776	MZ516021
MZ515616	MZ515852	MZ516015	MZ515599	MZ515779	MZ516022
MZ515617	MZ515854	MZ516017	MZ515603	MZ515785	MZ516025
MZ515618	MZ515859	MZ516024	MZ515605	MZ515786	MZ516030
MZ515619	MZ515860	MZ516026	MZ515607	MZ515793	MZ516041
MZ515620	MZ515861	MZ516027	MZ515608	MZ515794	MZ516042
MZ515628	MZ515862	MZ516028	MZ515610	MZ515807	MZ516049
MZ515629	MZ515866	MZ516029	MZ515612	MZ515809	MZ516051
MZ515631	MZ515875	MZ516031	MZ515613	MZ515812	MZ516054
MZ515632	MZ515876	MZ516033	MZ515615	MZ515813	MZ516056
MZ515634	MZ515877	MZ516038	MZ515625	MZ515817	MZ516060
MZ515640	MZ515878	MZ516039	MZ515627	MZ515818	MZ516061
MZ515643	MZ515881	MZ516040	MZ515636	MZ515820	MZ516062
MZ515645	MZ515882	MZ516043	MZ515637	MZ515823	MZ516065
MZ515647	MZ515884	MZ516044	MZ515638	MZ515824	MZ516081
MZ515649	MZ515887	MZ516047	MZ515639	MZ515827	MZ516095
MZ515650	MZ515896	MZ516048	MZ515653	MZ515829	MZ516098
MZ515651	MZ515898	MZ516052	MZ515656	MZ515830	MZ516102
MZ515652	MZ515901	MZ516053	MZ515658	MZ515831	MZ516104
MZ515655	MZ515902	MZ516057	MZ515660	MZ515832	MZ516105
MZ515679	MZ515905	MZ516058	MZ515663	MZ515836	MZ516107

RSV-A (N = 207)			RSV-B (N = 177)		
MZ515680	MZ515907	MZ516068	MZ515665	MZ515837	MZ516109
MZ515681	MZ515908	MZ516072	MZ515667	MZ515839	MZ516113
MZ515682	MZ515909	MZ516073	MZ515669	MZ515843	MZ516114
MZ515685	MZ515910	MZ516075	MZ515671	MZ515845	MZ516119
MZ515686	MZ515911	MZ516076	MZ515672	MZ515846	MZ516122
MZ515688	MZ515912	MZ516077	MZ515674	MZ515849	MZ516123
MZ515689	MZ515913	MZ516078	MZ515691	MZ515855	MZ516135
MZ515692	MZ515921	MZ516080	MZ515698	MZ515863	MZ516136
MZ515696	MZ515922	MZ516088	MZ515699	MZ515865	MZ516139
MZ515701	MZ515923	MZ516090	MZ515704	MZ515869	MZ516140
MZ515703	MZ515928	MZ516092	MZ515707	MZ515871	MZ516141
MZ515706	MZ515929	MZ516099	MZ515708	MZ515872	MZ516143
MZ515717	MZ515931	MZ516100	MZ515710	MZ515883	OP963385
MZ515718	MZ515933	MZ516103			
MZ515720	MZ515939	MZ516108			
MZ515722	MZ515940	MZ516110			
MZ515723	MZ515941	MZ516112			
MZ515731	MZ515942	MZ516117			
MZ515734	MZ515943	MZ516120			
MZ515740	MZ515945	MZ516129			
MZ515741	MZ515949	MZ516132			
MZ515744	MZ515951	MZ516134			
MZ515749	MZ515956	MZ516137			

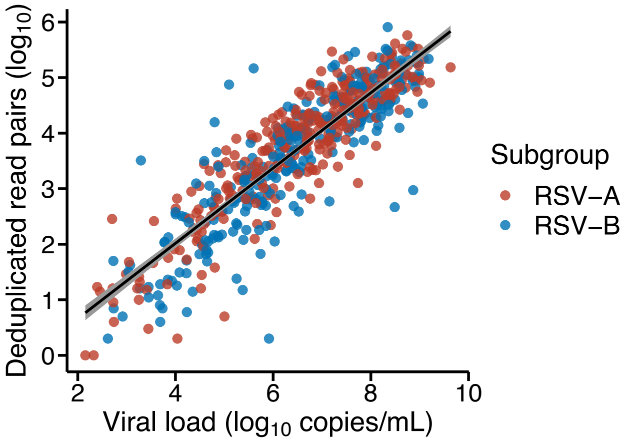

Supplementary Fig. 1 | Correlation between the number of unique RSV reads and RSV viral load (N = 578). Viral load data from reverse transcription quantitative PCR are available in 597 samples. Samples with both RSV subgroups detected were excluded from this figure (N = 9). Another 10 samples without any RSV reads recovered were also excluded from this figure. On average, for every log_{10} increase in read count, viral load increases by 1.2 log_{10}. Source data are provided as a Source Data file.

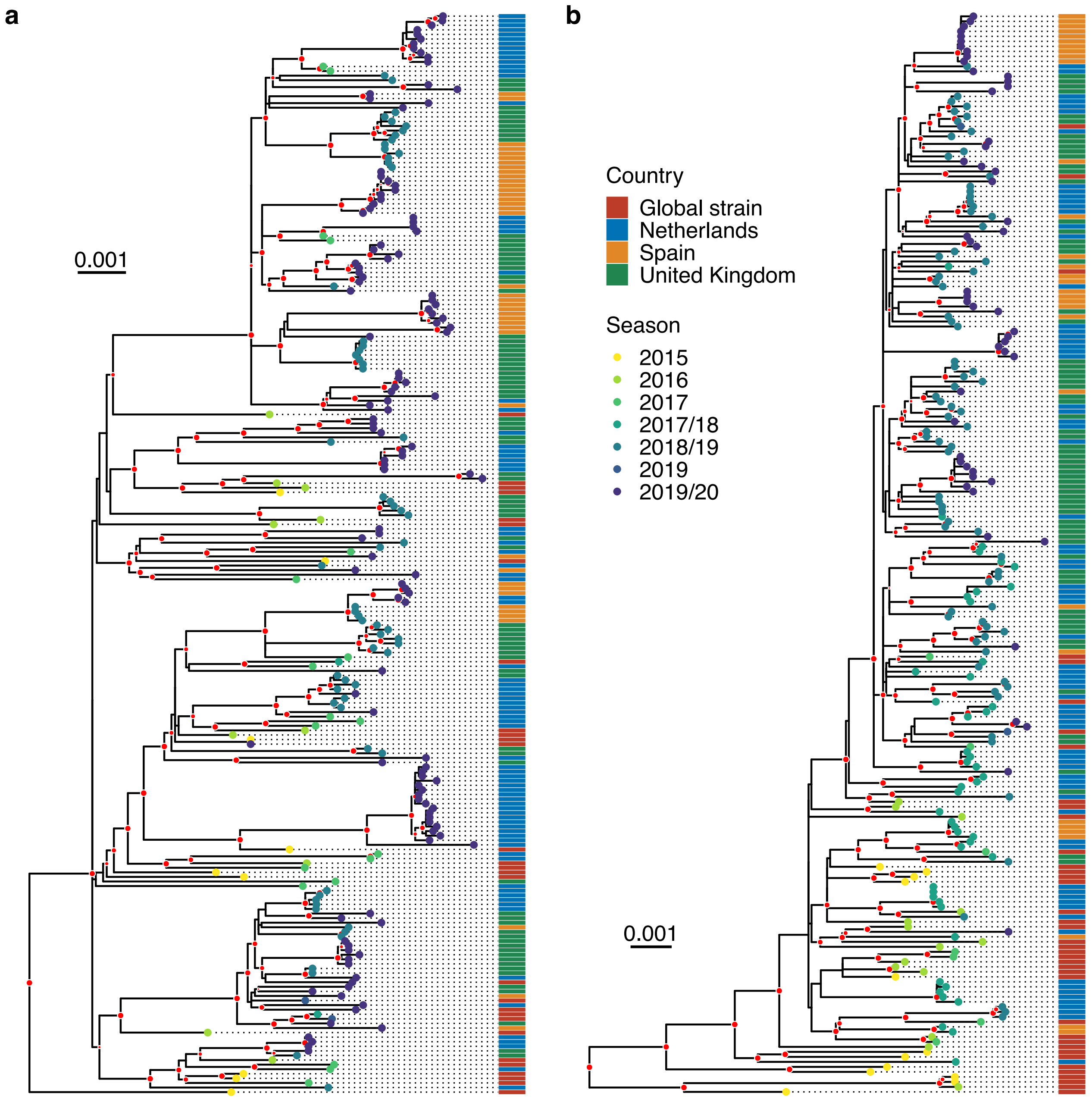

Supplementary Fig. 2 | Maximum-likelihood phylogenies. The phylogenies were reconstructed from the strains in the present study and strains collected from all over the world between 2015 and 2019, downloaded from GenBank. **a** RSV-A phylogeny (N = 236). All RSV-A strains from the present study (N = 207) and GenBank (N = 29) were genotype ON1, except for the one at the very bottom, which is genotype GA2, downloaded from GenBank (accession number MH181907). **b** RSV-B phylogeny (N = 216). All RSV-B strains from the present study (N = 177) and GenBank (N = 39) were genotype BA. On average, ON1 strains had greater patristic distances than BA ones (mean ± standard deviation, 0.0100 ± 0.0028 vs. 0.0063 ± 0.0030; two-tailed Mann–Whitney U test, $P < 2.2 \times 10^{-16}$), whereas BA strains showed stronger temporal clustering than ON1 ones. The phylogenies were inferred using RAxML with the general time reversible nucleotide substitution model and gamma-distributed rate heterogeneity among sites. The sampling season and country of each strain are illustrated by tip and bar colours, respectively. Strains labelled as Netherlands, Spain, and United Kingdom were generated from this study, and the remainder were downloaded from GenBank. Red dots show well-supported nodes with a bootstrap value over 70% and are sized in proportion to bootstrap values. The trees were rooted to the oldest strains sampled during 1962–1977—GenBank accession numbers KU316149 and KU316166 for RSV-A, and KU316116 and MG813995 for RSV-B. These oldest strains are not shown here.

The scale bars represent the number of nucleotide substitutions per site. Source data are provided as Source Data files.

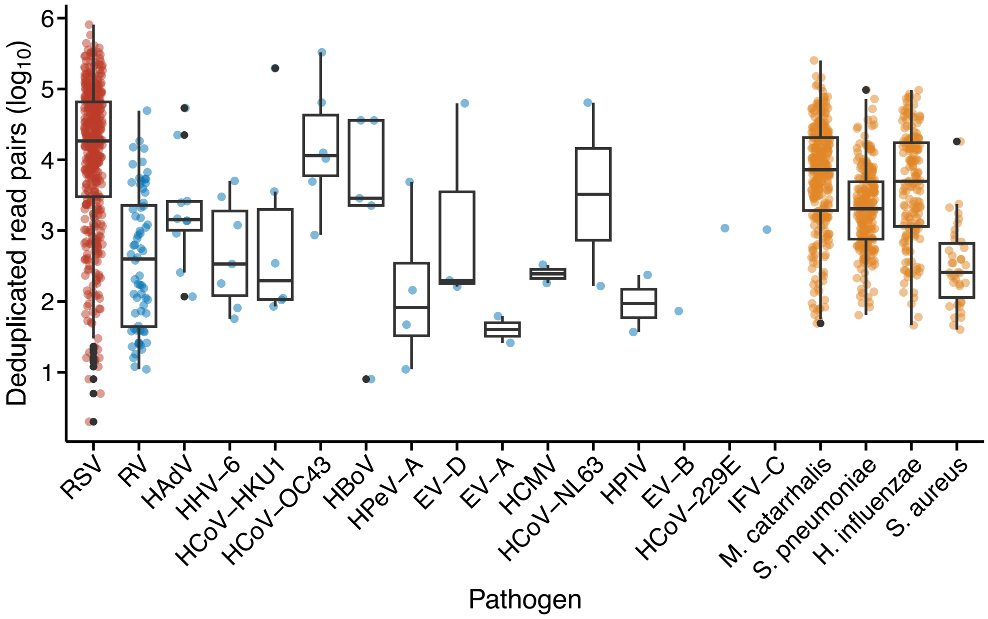

Supplementary Fig. 3 | Number of deduplicated read pairs mapped to each co-detected pathogen in this study. The centre line of each box denotes the median; box limits, the first and third quartiles; whiskers, the highest and lowest values within 1.5 times the interquartile range from the box limits; and outlying points, outliers. The species from left to right are: respiratory syncytial virus (RSV; N = 433), rhinovirus (RV; N = 68), human adenovirus (HAdV; N = 10), human herpesvirus 6 (HHV-6; N = 7), human coronavirus HKU1 (HCoV-HKU1; N = 6), human coronavirus OC43 (HCoV-OC43; N = 6), human bocavirus (HBoV; N = 5), human parechovirus A (HPeV-A; N = 4), enterovirus D (EV-D; N = 3), enterovirus A (EV-A; N = 2), human cytomegalovirus (HCMV; N = 2), human coronavirus NL63 (HCoV-NL63; N = 2), human parainfluenza virus (HPIV; N = 2), enterovirus B (EV-B; N = 1), human coronavirus 229E (HCoV-229E; N = 1), influenza C virus (IFV-C; N = 1), *Moraxella catarrhalis* (N = 286), *Streptococcus pneumoniae* (N = 222), *Haemophilus influenzae* (N = 164), and *Staphylococcus aureus* (N = 38). Source data are provided as a Source Data file.

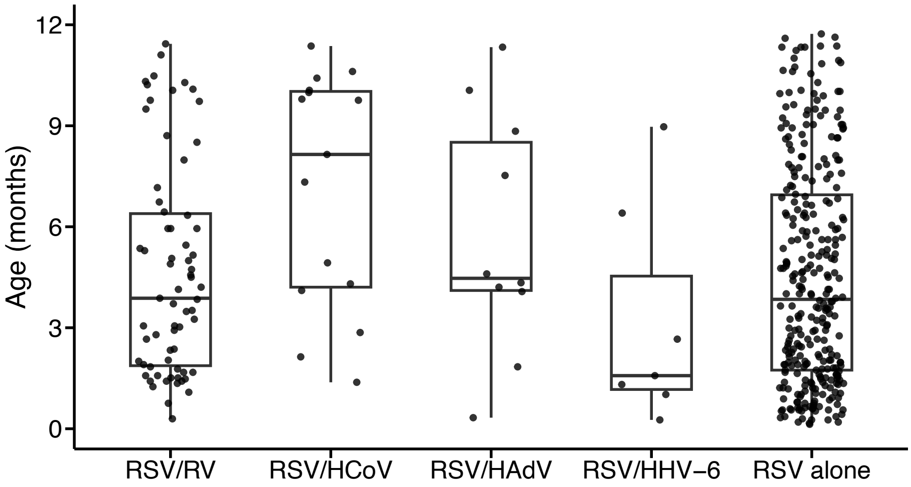

Supplementary Fig. 4 | Age distribution among RSV-infected infants with each co-detected virus or with RSV alone. The number of infants with respiratory syncytial virus (RSV)/rhinovirus (RV), RSV/human coronavirus (HCoV), RSV/human adenovirus (HAdV), RSV/human herpesvirus 6 (HHV-6), and RSV alone was 68, 15, 10, 7, and 307, respectively. The median (interquartile range) age of infants with RSV/RV, RSV/HCoV, RSV/HAdV, RSV/HHV-6, and RSV alone was 3.9 (1.9–6.4), 8.1 (4.2–10.0), 4.5 (4.1–8.5), 1.6 (1.2–4.5), and 3.8 (1.7–6.9) months, respectively. The age distribution between these groups was significantly different (Kruskal–Wallis test, P = 0.025). Infants with RSV/HCoV co-detection were significantly older than those with RSV/HHV-6 co-detection and those with RSV alone (two-sided Dunn's post hoc test with the Benjamini–Hochberg method, adjusted P = 0.033 and 0.046, respectively). The centre line of each box denotes the median; box limits, the first and third quartiles; and whiskers, the highest and lowest values within 1.5 times the interquartile range from the box limits. Source data are provided as a Source Data file.

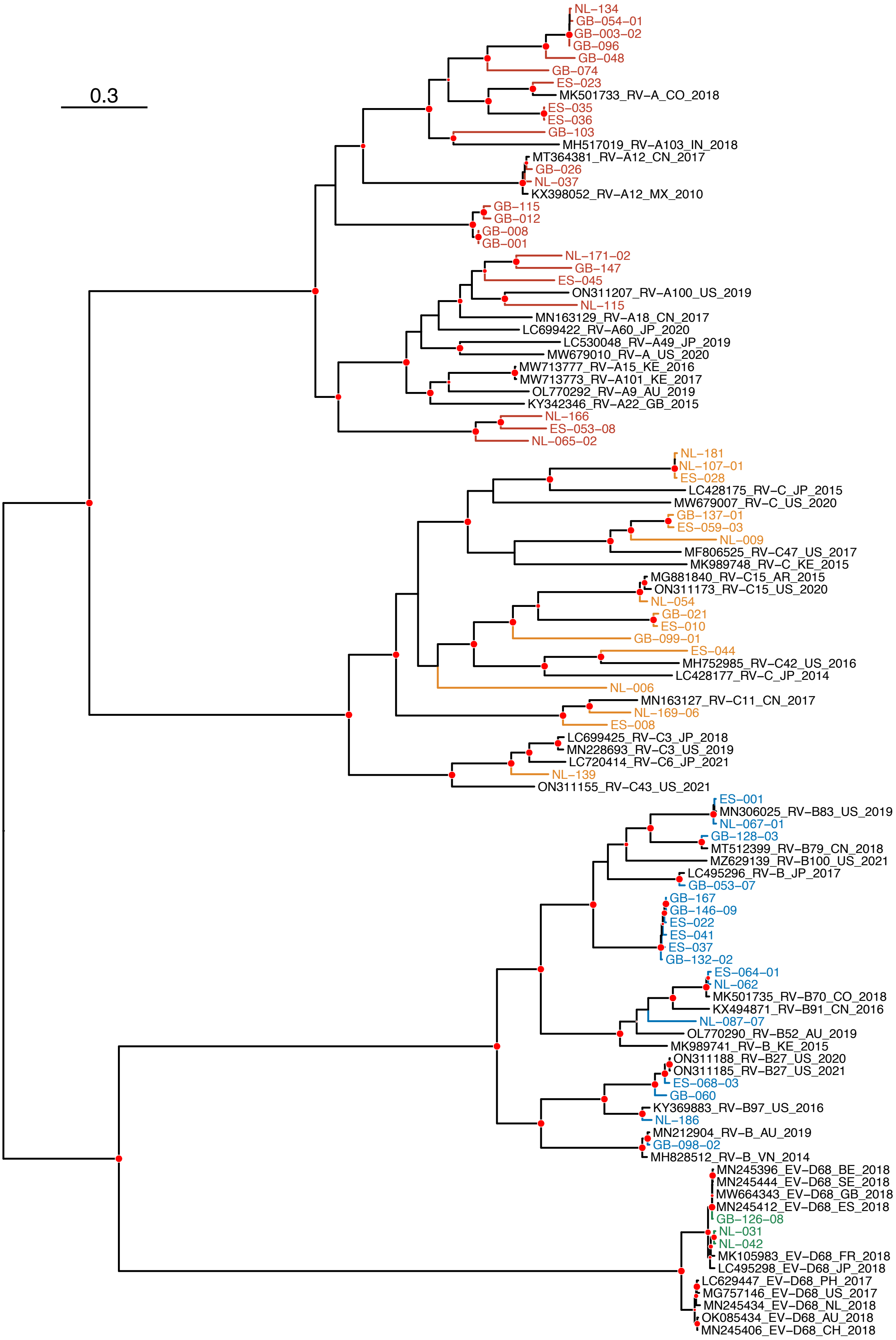

Supplementary Fig. 5 | Phylogeny of genus *Enterovirus*. Samples where less than 50% of the genome was recovered were removed from the reconstruction. For infants who had multiple samples collected, only the sample with the highest coverage of the *Enterovirus* genome was included. The samples from this study (N = 58) were further supplemented with 50 complete or nearly complete *Enterovirus* genomes downloaded from GenBank, collected from across the globe between 2010 and 2021. RAxML was used to reconstruct the

maximum-likelihood phylogeny with the general time reversible nucleotide substitution model and gamma-distributed rate heterogeneity among sites. The tree was midpoint rooted. Samples from this study are shown in different colours: *Rhinovirus A* (red), *Rhinovirus B* (blue), *Rhinovirus C* (tangerine), and *Enterovirus D* (green). All *Enterovirus D* strains were enterovirus D68. Samples were labelled as anonymised subject ID (two country letters followed by three digits) and order of serial samples if applicable. Strains downloaded from GenBank are shown in black, labelled as GenBank accession number, followed by type, sampling country, and sampling year. Red dots show well-supported nodes with a bootstrap value over 70% and are sized in proportion to bootstrap values. The scale bar represents the number of nucleotide substitutions per site. Source data are provided as Source Data files.
Abbreviations: *AR* Argentina, *AU* Australia, *BE* Belgium, *CH* Switzerland, *CN* China, *CO* Colombia, *ES* Spain, *FR* France, *GB* United Kingdom, *IN* India, *JP* Japan, *KE* Kenya, *MX* Mexico, *NL* Netherlands, *PH* Philippines, *SE* Sweden, *US* United States, *VN* Viet Nam.

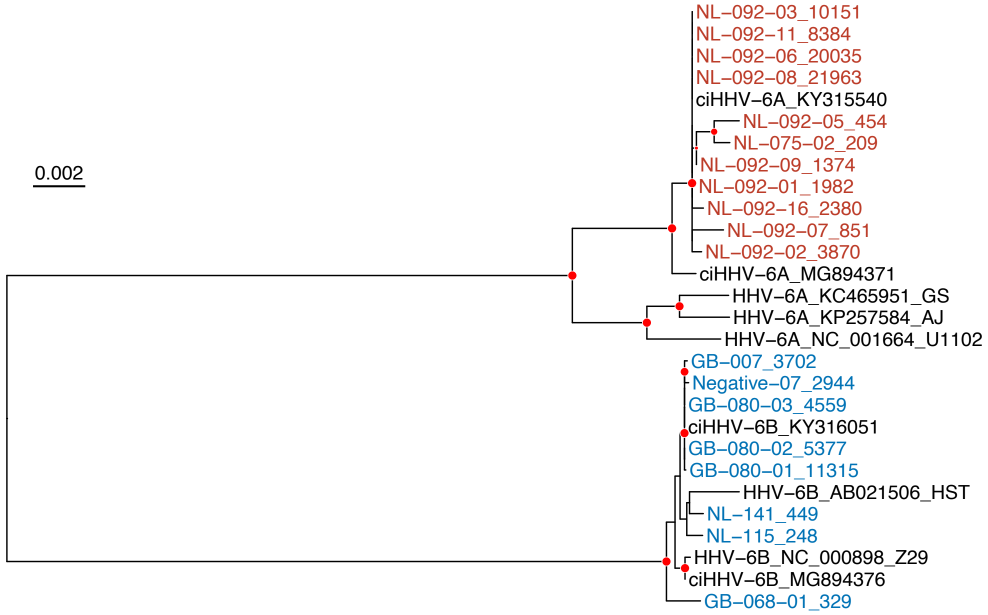

Supplementary Fig. 6 | Phylogeny of human herpesvirus 6 (HHV-6). RAxML was used to reconstruct the maximum-likelihood phylogeny with the general time reversible nucleotide substitution model and gamma-distributed rate heterogeneity among sites. Coloured taxa are samples collected from this study (N = 19), with HHV-6A in red and HHV-6B in blue. They are labelled as anonymised patient ID (two country letters followed by three digits), order of serial samples if applicable, and number of HHV-6 reads, except for one taxon labelled as Negative-07_2944, which had 2,944 HHV-6 reads and was collected from an infant who had respiratory symptoms but tested negative for RSV. Taxa in black represent the GenBank reference genomes (N = 9), labelled as HHV-6 type (ci denotes chromosomally integrated), GenBank accession number, and strain name if available. The tree was midpoint rooted. Red dots show well-supported nodes with a bootstrap value over 70% and are sized in proportion to bootstrap values. The scale bar represents the number of nucleotide substitutions per site. Source data are provided as Source Data files.

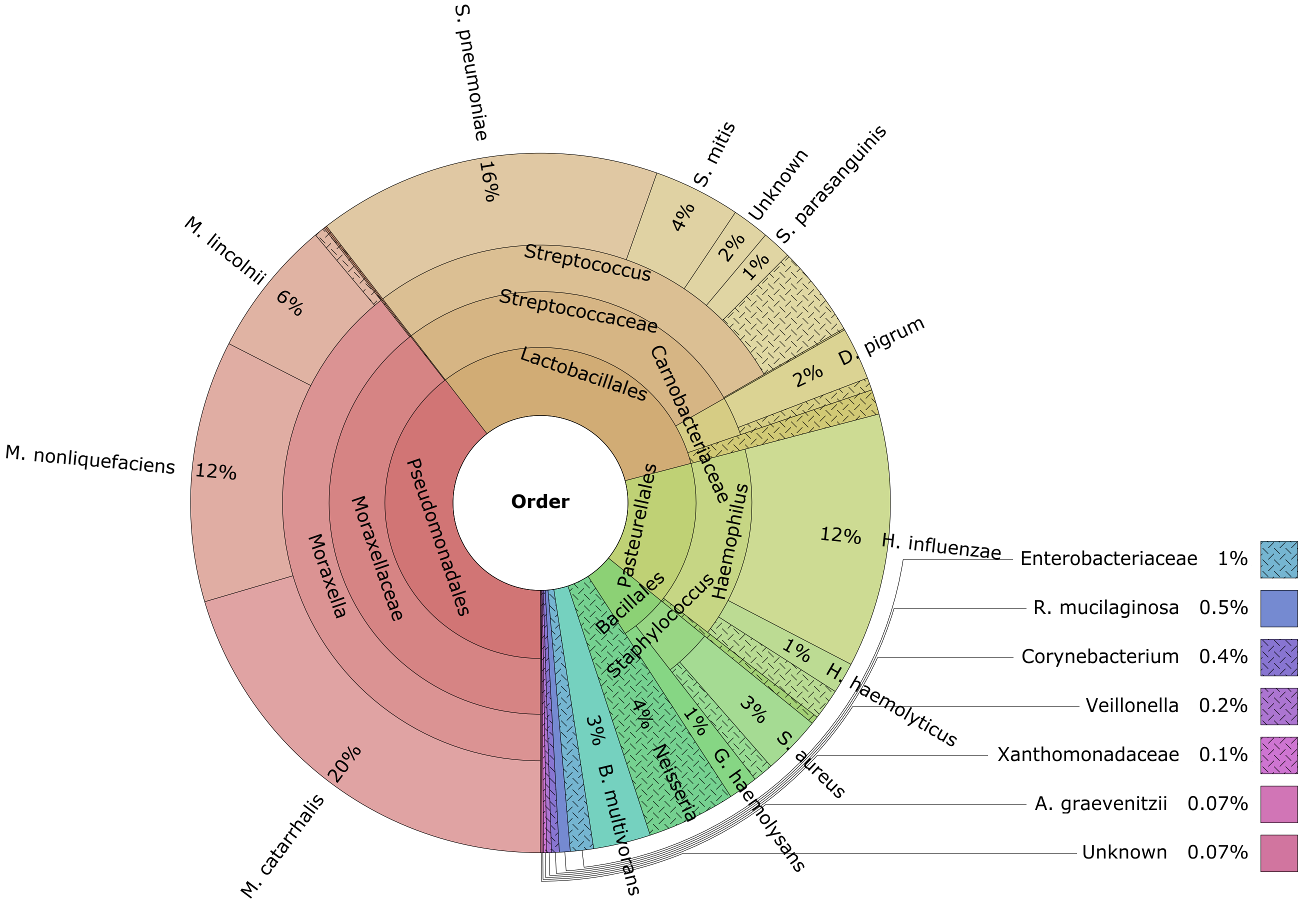

Supplementary Fig. 7 | Composition of co-detected bacteria in RSV-infected infants (N = 433). The percentage of a bacterial species was calculated as the number of infants having this bacterial species divided by the sum of the number of bacterial species found in each infant. Circles from inside to outside represent the order, family, genus, and species of the bacteria with some levels collapsed for better visualisation. Interactive visualisation via HTML can be accessed in Supplementary Data 1. Source data are provided as a Source Data file.

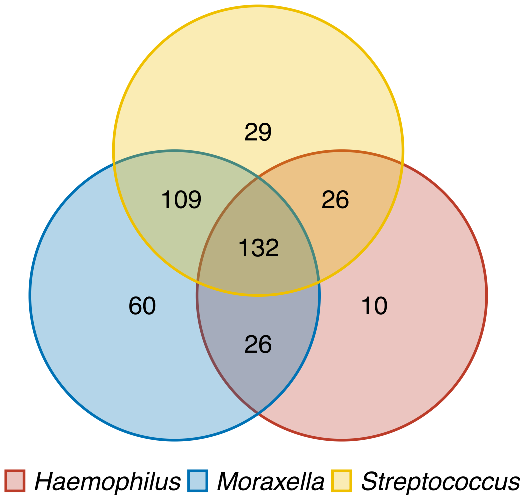

Supplementary Fig. 8 | Venn diagram of the top three co-detected bacterial genera in RSV-infected infants (N = 433). The number in each area represents the number of infants. Forty-one infants did not have any of the three bacterial genera. Source data are provided as a Source Data file.

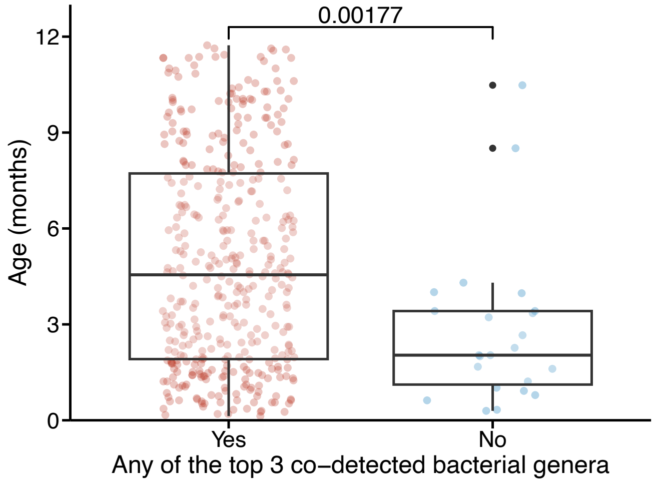

Supplementary Fig. 9 | Age distribution among infants with and without any of the top three co-detected bacterial genera (N = 413). These bacterial genera were *Moraxella*, *Streptococcus*, and *Haemophilus*, at least one of which was detected in 91% (392/433) of the infants. In contrast, 5% (23/433) of the infants did not have any of the bacterial genera recovered, and 4% (18/433) of the infants had only an equivocal presence of any of the bacterial genera, that were therefore excluded from this comparison. An additional two infants (with any of the three genera) were also excluded from this comparison since they did not have the age information available. The centre line of each box denotes the median; box limits, the first and third quartiles; whiskers, the highest and lowest values within 1.5 times the interquartile range from the box limits; and outlying points, outliers. Coloured overlaid points represent each individual infants. A two-tailed Mann–Whitney U-test was used to evaluate the difference with the P value shown above the plot. Source data are provided as a Source Data file.

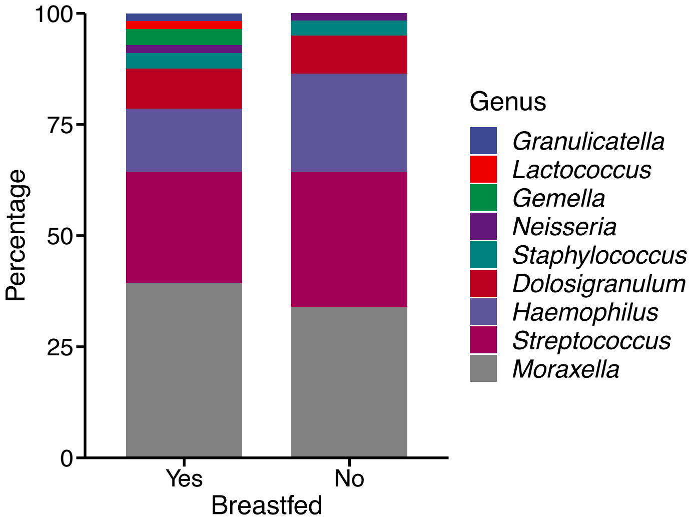

Supplementary Fig. 10 | Co-detected bacterial genera by breastfeeding status (N = 51). Breastfed was defined as either exclusively breastfed or in combination of formula milk within the 4 weeks prior to the RSV infection. At least one bacterial genus was found in 25/27 (93%) breastfed and 24/24 (100%) exclusively formula-fed infants. A mean of 2.1 and 2.5 bacterial genera were identified in each breastfed and exclusively formula-fed infants, respectively, and this difference was not significant (two-tailed Mann–Whitney U test, P = 0.115). Source data are provided as a Source Data file.

a

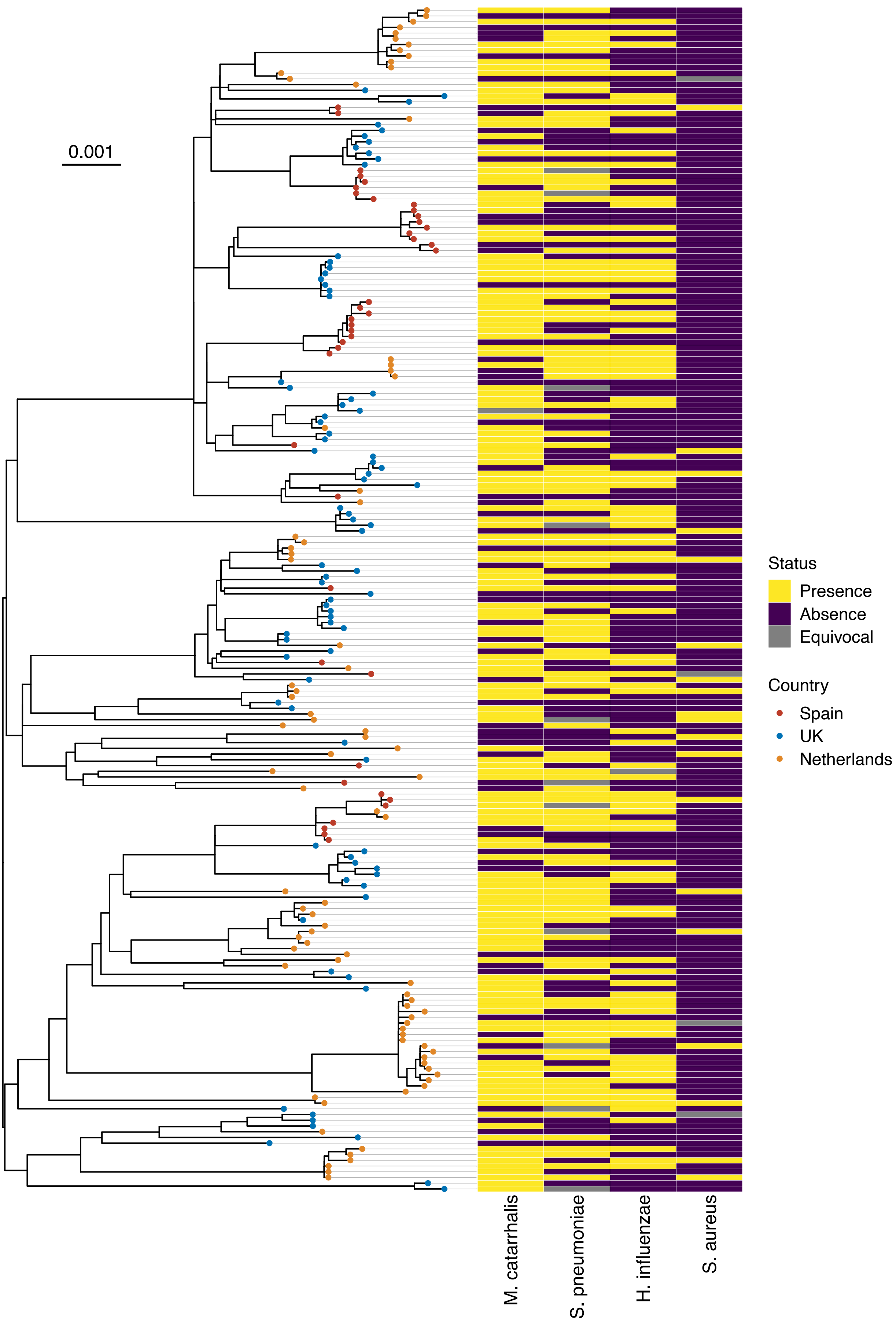
0.001
M. catarrhalis
S. pneumoniae
H. influenzae
S. aureus
Status
Presence
Absence
Equivocal
Country
Spain
UK
Netherlands

b

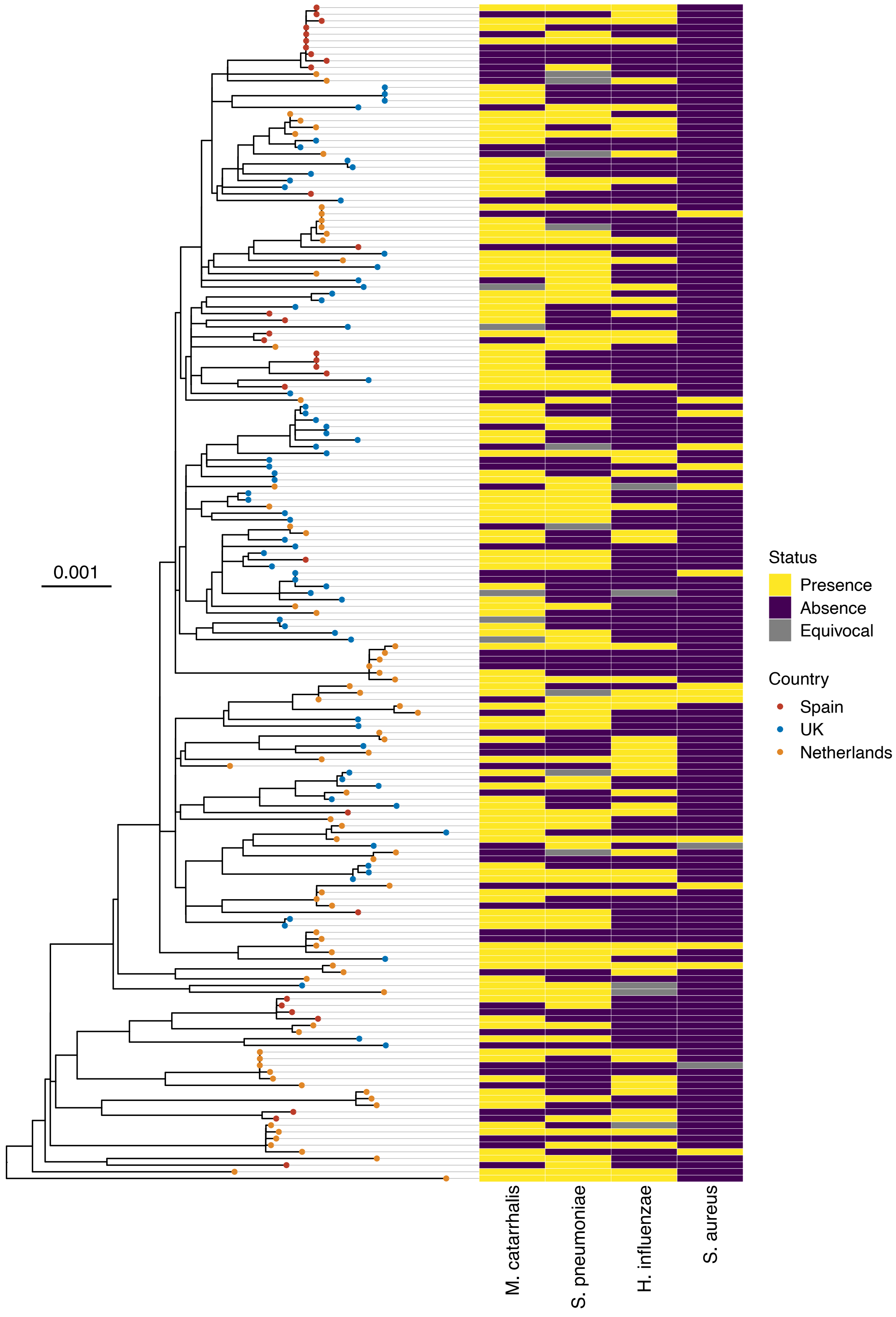
0.001
M. catarrhalis
S. pneumoniae
H. influenzae
S. aureus
Status
Presence
Absence
Equivocal
Country
Spain
UK
Netherlands

Supplementary Fig. 11 | Distribution of commonly co-detected bacteria on the RSV maximum-likelihood phylogenies. a RSV-A phylogeny was reconstructed from 207 samples. **b** RSV-B phylogeny was reconstructed from 177 samples. Only samples with at least 70% of the coding sequences recovered were included. For infants with multiple samples collected, only the sample with the highest coverage was included. The phylogenetic trees were reconstructed using RAxML with the general time reversible nucleotide substitution model and gamma-distributed rate heterogeneity among sites. The trees were midpoint rooted. The sampling country of each strain is illustrated by tip colour. The scale bars represent the number of nucleotide substitutions per site. Source data are provided as Source Data files.
Abbreviations: *M. catarrhalis*, *Moraxella catarrhalis*; *S. pneumoniae*, *Streptococcus pneumoniae*; *H. influenzae*, *Haemophilus influenzae*; *S. aureus*, *Staphylococcus aureus*.

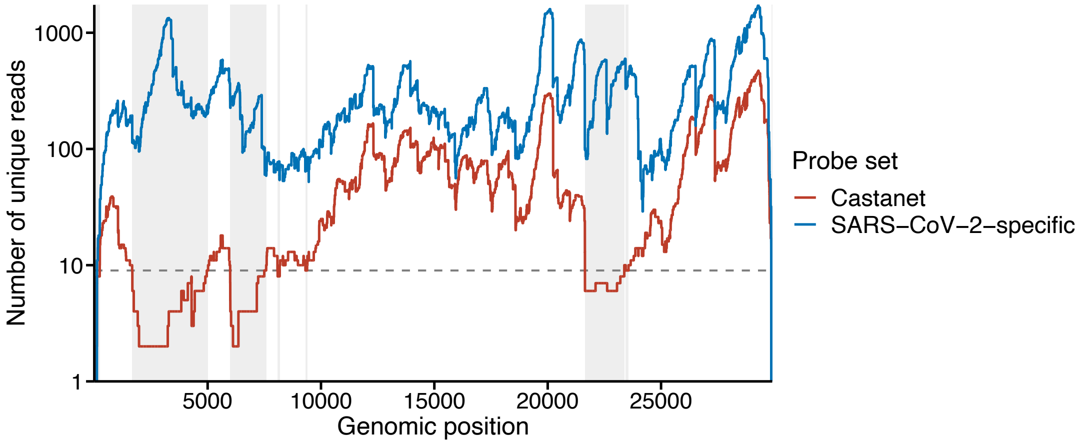

Supplementary Fig. 12 | Coverage plot for SARS-CoV-2. In a previous study,[1] a sample collected from a patient infected with severe acute respiratory syndrome coronavirus 2 (SARS-CoV-2) was sequenced using targeted metagenomic sequencing with the *Castanet* probe set (red) and a SARS-CoV-2-specific probe set (blue). The SARS-CoV-2 genome was reconstructed as previously described.[1] Genomic regions with coverage of <10 *Castanet*-captured unique reads were shaded in grey (7.3 kb). The dashed grey line represents nine unique reads. Source data are provided as a Source Data file.

Supplementary Reference

1. Lythgoe, K. A. et al. SARS-CoV-2 within-host diversity and transmission. *Science* **372,** eabg0821 (2021).
