# Supplementary material for: Targeted metagenomics reveals association between severity and pathogen co-detection in infants with respiratory syncytial virus
Source: Nat Commun. 2024 Mar 16;15:2379. doi: 10.1038/s41467-024-46648-3 (PMC10944482; doi:10.1038/s41467-024-46648-3)
Supplement: Supplementary file 3 — Description of Additional Supplementary Files [file 41467_2024_46648_MOESM3_ESM.pdf]

Description of Additional Supplementary Files

Supplementary Data 1. Interactive Krona chart via HTML to visualise the composition of co-detected bacteria in the RSV-infected infants (N = 433). The percentage of a bacterial species was calculated as the number of infants having this bacterial species divided by the sum of the number of bacterial species found in each infant. Circles from inside to outside represent the order, family, genus, and species of the bacteria.
