# Supplementary material for: Targeted metagenomics reveals association between severity and pathogen co-detection in infants with respiratory syncytial virus
Source: Nat Commun. 2024 Mar 16;15:2379. doi: 10.1038/s41467-024-46648-3 (PMC10944482; doi:10.1038/s41467-024-46648-3)
Supplement: Supplementary file 4 — Supplementary Data 1 [file 41467_2024_46648_MOESM4_ESM.html]

Javascript must be enabled to view this page.

magnitude

Infant

 1399

 553

 552

 551

 286

 169

 89

 7

 1

 1

 1

 1

 1

 439

 381

 380

 222

 56

 24

 19

 14

 12

 12

 7

 5

 3

 2

 1

 1

 1

 1

 1

 1

 42

 34

 34

 8

 6

 2

 16

 16

 14

 2

 209

 209

 204

 164

 20

 14

 3

 3

 3

 3

 2

 2

 70

 51

 51

 38

 9

 2

 1

 1

 19

 19

 19

 37

 37

 37

 37

 57

 57

 57

 18

 15

 9

 8

 3

 2

 2

 7

 7

 7

 7

 15

 15

 5

 5

 5

 5

 2

 2

 1

 1

 2

 1

 1

 5

 5

 5

 2

 1

 1

 1

 3

 3

 3

 2

 1

 1

 1

 1

 1

 1

 1

 1

 1

 2

 2

 1

 1

 1

 1
