# Supplementary material for: Targeted metagenomics reveals association between severity and pathogen co-detection in infants with respiratory syncytial virus
Source: Nat Commun. 2024 Mar 16;15:2379. doi: 10.1038/s41467-024-46648-3 (PMC10944482; doi:10.1038/s41467-024-46648-3)
Supplement: Supplementary file 5 — Reporting Summary [file 41467_2024_46648_MOESM5_ESM.pdf]

nature portfolio

Corresponding author(s): Gu-Lung Lin, Tanya Golubchik

Last updated by author(s): Feb 8, 2024

Reporting Summary

Nature Portfolio wishes to improve the reproducibility of the work that we publish. This form provides structure for consistency and transparency in reporting. For further information on Nature Portfolio policies, see our Editorial Policies and the Editorial Policy Checklist.

Statistics

For all statistical analyses, confirm that the following items are present in the figure legend, table legend, main text, or Methods section.

n/a	Confirmed	
☐	☒	The exact sample size (n) for each experimental group/condition, given as a discrete number and unit of measurement
☐	☒	A statement on whether measurements were taken from distinct samples or whether the same sample was measured repeatedly
☐	☒	The statistical test(s) used AND whether they are one- or two-sided<br>*Only common tests should be described solely by name; describe more complex techniques in the Methods section.*
☐	☒	A description of all covariates tested
☐	☒	A description of any assumptions or corrections, such as tests of normality and adjustment for multiple comparisons
☐	☒	A full description of the statistical parameters including central tendency (e.g. means) or other basic estimates (e.g. regression coefficient) AND variation (e.g. standard deviation) or associated estimates of uncertainty (e.g. confidence intervals)
☐	☒	For null hypothesis testing, the test statistic (e.g. F, t, r) with confidence intervals, effect sizes, degrees of freedom and P value noted<br>*Give P values as exact values whenever suitable.*
☒	☐	For Bayesian analysis, information on the choice of priors and Markov chain Monte Carlo settings
☒	☐	For hierarchical and complex designs, identification of the appropriate level for tests and full reporting of outcomes
☐	☒	Estimates of effect sizes (e.g. Cohen's d, Pearson's r), indicating how they were calculated

Our web collection on statistics for biologists contains articles on many of the points above.

Software and code

Policy information about availability of computer code

Data collection	Electronic data capture (EDC) systems: REDCap and OpenClinica
Data analysis	BBMap (version 2020-02-13; https://sourceforge.net/projects/bbmap/): removal of human reads<br>Bowtie 2 (version 2.4.1): read mapping<br>Castanet (https://github.com/tgolubch/castanet): determination of genomic coverage of targeted pathogens<br>Enterovirus Typing Tool (https://www.rivm.nl/mpf/typingtool/enterovirus/): enterovirus genotyping<br>GenBank: source of global viral strains included in the phylogenetic analysis<br>IVA (version 1.0.8): de novo assembly<br>Kraken 2 (version 0.39): taxonomic classification<br>Krona (https://github.com/marbl/Krona/wiki): interactive metagenomic visualisation<br>mafft (version 7.490): multiple sequence alignment<br>MarkDuplicates (Picard tools, version 2.18.14): removal of duplicated reads<br>metaSPAdes (version 3.14.1): de novo assembly<br>PubMLST database and its RESTful application programming interface (https://pubmlst.org/species-id): identification of matching bacterial species<br>R (version 4.3.1): statistical analysis and figure generation<br>R code used for statistical analysis in the tables: https://doi.org/10.5281/zenodo.10626081 (licensed under a Creative Commons Attribution 4.0 International License)<br>RAxML (version 8.2.12): maximum-likelihood phylogenetic analysis<br>shiver (https://github.com/ChrisHIV/shiver; downloaded on 2020-08-13): genome reconstruction

Trimmomatic (version 0.39): adapter and quality trimming

For manuscripts utilizing custom algorithms or software that are central to the research but not yet described in published literature, software must be made available to editors and reviewers. We strongly encourage code deposition in a community repository (e.g. GitHub). See the Nature Portfolio guidelines for submitting code & software for further information.

Data

Policy information about availability of data

All manuscripts must include a data availability statement. This statement should provide the following information, where applicable:

- Accession codes, unique identifiers, or web links for publicly available datasets
- A description of any restrictions on data availability
- For clinical datasets or third party data, please ensure that the statement adheres to our policy

Sequencing data generated in this study have been deposited in the European Nucleotide Archive with the accession code PRJEB34042 (https://www.ebi.ac.uk/ena/data/view/PRJEB34042). The RSV consensus sequences included in the phylogenetic analyses have been deposited in GenBank with the accession numbers shown in Supplementary Table 19. The PubMLST multi-species isolate database, integrating curated allelic and bacterial species information, used to identify bacterial species, is available at https://pubmlst.org/species-id. Source data are provided with this paper, with patients' age shown in 30-day increments and gestational age shown as term/preterm to prevent the identification of individuals.

Research involving human participants, their data, or biological material

Policy information about studies with human participants or human data. See also policy information about sex, gender (identity/presentation), and sexual orientation and race, ethnicity and racism.

Reporting on sex and gender	This study analysed samples and data collected from participants of both sexes. Participants were enrolled into the clinical studies regardless of their sex. Sex was determined following external examination of body characteristics. This study reported data from 192 females, 239 males, and two participants with missing information on sex.<br>No sex-based analysis was performed as this was not our research question. However, sex was one of the covariates and was adjusted for in statistical analysis.<br>Disaggregated data on sex have been provided in the Source Data.
Reporting on race, ethnicity, or other socially relevant groupings	This study did not report or use any socially constructed or socially relevant categorisation variables.
Population characteristics	The covariate-relevant population characteristics of the participants (N = 433) in this study included age, gestational age, sex, presence of comorbidity, and subgroup of respiratory syncytial virus (RSV).<br>Median age: 4.1 months (interquartile range: 1.9–7.5 months) (Two participants had missing information on age.)<br>Median gestational age: 39.6 weeks (interquartile range: 38.6–40.4 weeks) (Two participants had missing information on gestational age.)<br>Female sex: 192/431 (45%) (Two participants had missing information on sex.)<br>Presence of comorbidity: 37/432 (9%) (One participant had missing information on comorbidity.)<br>RSV subgroup A infection: 220/424 (52%) (Nine participants had both RSV subgroups A and B identified in their samples.)
Recruitment	The parents or guardians of potential participants of the clinical studies were approached by study staff in the community, emergency departments, or hospital wards.<br>Only samples from infants with at least one sample where RSV sequencing was possible were included in this study (N = 831). This might have introduced a selection bias because samples where RSV sequencing was impossible may be samples with a very low RSV viral load collected from participants with certain features. However, among all sequenced samples, only eight samples were excluded from the analysis. In addition, this sequencing method was sensitive and the included dataset encompassed participants with a wide range of RSV viral load and disease severity, so the results were representative of the tested population.
Ethics oversight	The clinical study protocols were approved by the relevant authorities and ethics committees at each site: Hospital Clínico Universitario de Santiago de Compostela, Comité de Ética de la Investigación de Santiago-Lugo (no. 2017/395) in Spain; the University of Oxford, the Health Research Authority (no. 231136), the NHS National Research Ethics Service Oxfordshire Research Ethics Committee A (no. 15/SC/0335) and South Central – Hampshire A (no. 17/SC/0522) in the UK; and the Medical Ethical Committee, University Medical Center Utrecht (no. 17/563) in the Netherlands.<br>The parents or guardians of all participants provided written, informed consent. Reimbursement was only offered where the participant had to travel to the clinic site for a study visit.

Note that full information on the approval of the study protocol must also be provided in the manuscript.

Field-specific reporting

Please select the one below that is the best fit for your research. If you are not sure, read the appropriate sections before making your selection.

[x] Life sciences [ ] Behavioural & social sciences [ ] Ecological, evolutionary & environmental sciences

For a reference copy of the document with all sections, see nature.com/documents/nr-reporting-summary-flat.pdf

Life sciences study design

All studies must disclose on these points even when the disclosure is negative.

Sample size	No sample-size calculation was performed in this study. All RSV-positive nasopharyngeal swabs (N = 839) collected from the two clinical studies were sequenced. All samples from infants with at least one sample where sequencing was successful and RSV reads were recovered were included in the study analyses (N = 831). These 831 samples were collected from 433 participants.
Data exclusions	Seven participants and their eight samples were excluded from the study analysis because all eight samples were either sequencing failure or without any RSV reads recovered. The exclusion criteria were pre-established as we aimed to investigate co-detected pathogens in participants with documented RSV infection.
Replication	Our study was based on 831 RSV samples (i.e., biological replicates), and thus the findings presented in the manuscript were summarised from these 831 replicates. No samples were sequenced repeatedly. All data analyses were replicated at least twice using the R code provided in the 'Code Availability' statement. All attempts at replication of the data analysis were successful.
Randomization	All available RSV-positive samples were sequenced, so no allocation or randomisation was performed.
Blinding	Investigators were not blinded during data collection and analysis. Blinding was not relevant to our study as there was no group allocation involved in this study.

Reporting for specific materials, systems and methods

We require information from authors about some types of materials, experimental systems and methods used in many studies. Here, indicate whether each material, system or method listed is relevant to your study. If you are not sure if a list item applies to your research, read the appropriate section before selecting a response.

Materials & experimental systems

n/a	Involved in the study
☒	☐ Antibodies
☒	☐ Eukaryotic cell lines
☒	☐ Palaeontology and archaeology
☒	☐ Animals and other organisms
☐	☒ Clinical data
☒	☐ Dual use research of concern
☒	☐ Plants

Methods

n/a	Involved in the study
☒	☐ ChIP-seq
☒	☐ Flow cytometry
☒	☐ MRI-based neuroimaging

Clinical data

Policy information about clinical studies

All manuscripts should comply with the ICMJEguidelines for publication of clinical research and a completedCONSORT checklist must be included with all submissions.

Clinical trial registration	Longitudinal birth cohort study: NCT03627572<br>Infant cross-sectional study: NCT03756766
Study protocol	Longitudinal birth cohort study: https://doi.org/10.1093/infdis/jiaa310<br>Infant cross-sectional study: https://doi.org/10.1093/infdis/jiaa239
Data collection	Participants were enrolled from the community, emergency departments, or hospital wards where their data and samples were collected. The studies were conducted in Spain, the UK, and the Netherlands. The recruitment period was during 2017–2020.
Outcomes	Genetic characteristics of RSV and co-detected pathogens were one of the predefined secondary outcomes of the clinical studies. Next-generation sequencing of nasopharyngeal swabs with target enrichment and bioinformatic analysis of the sequencing data were carried out to assess these measures.
